# Engineering of the *XY* Magnetic Layered System with Adeninium Cations: Monocrystalline Angle-Resolved Studies of Nonlinear Magnetic Susceptibility

**DOI:** 10.1021/acs.inorgchem.1c00432

**Published:** 2021-07-07

**Authors:** Emilia Kuzniak-Glanowska, Piotr Konieczny, Robert Pełka, Tadeusz M. Muzioł, Marcin Kozieł, Robert Podgajny

**Affiliations:** † Faculty of Chemistry, Jagiellonian University, Gronostajowa 2, 30-387 Kraków, Poland; ‡ Institute of Nuclear Physics PAN, Radzikowskiego 152, 31-342 Kraków, Poland; § Faculty of Chemistry, Nicolaus Copernicus University in Torun, Gagarina 7, 87-100 Torun, Poland

## Abstract

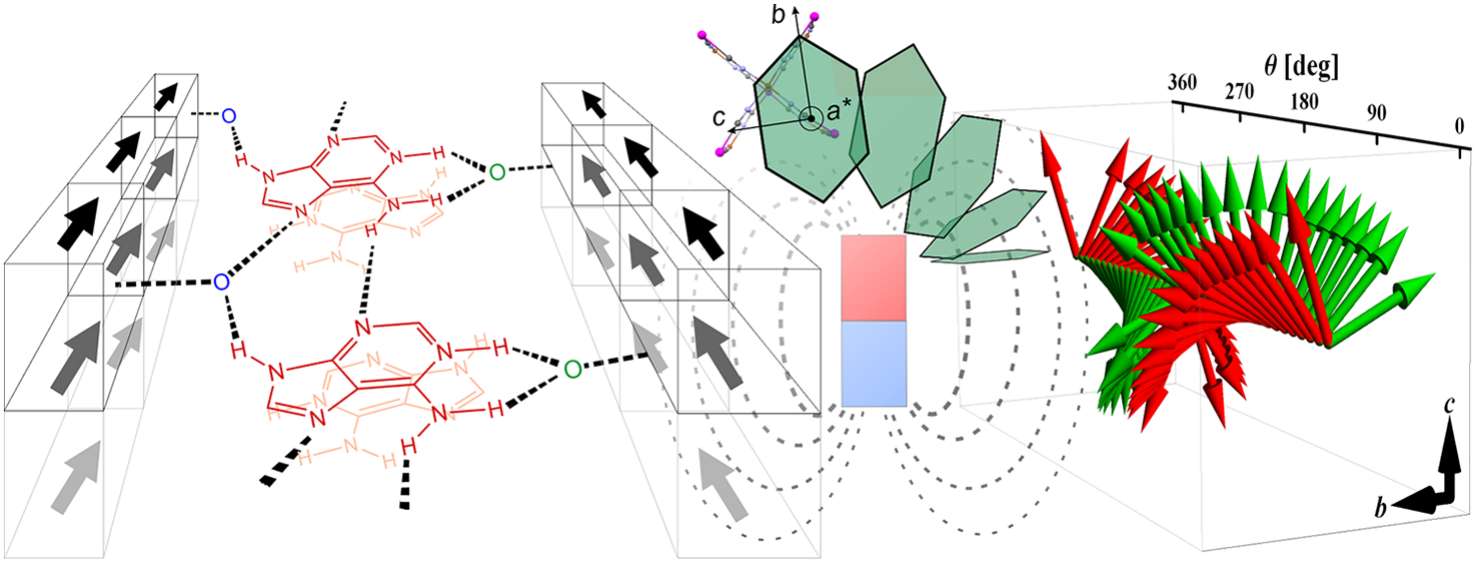

An original example of *modular* crystal engineering involving molecular magnetic {Cu^II^[W^V^(CN)_8_]}^−^ bilayers and adeninium cations (AdeH^+^) toward the new layered molecular magnet (AdeH){Cu^II^[W^V^(CN)_8_]}·2H_2_O (**1**) is presented. **1** crystallizes within the monoclinic *C*2 space group (*a* = 41.3174(12), *b* = 7.0727(3), *c* = 7.3180(2) Å, β = 93.119(3)°, and *V* = 2135 Å^3^). The bilayer topology is based on a stereochemical matching between the square pyramidal shape of Cu^II^ moiety and the bicapped trigonal prismatic shape of [W^V^(μ-CN)_5_(CN)_3_], and the separation between bilayers is significantly increased (by ∼50%; from ca. 9.5 to ca. 14.5 Å) compared to several former analogues in this family. This was achieved via a unique combination of (i) a 1D ribbonlike hydrogen bond system {AdeH^+^···H_2_O···AdeH^+^···}_∞_ exploiting planar water-assisted Hoogsteen···Sugar synthons with (ii) parallel 1D π–π stacks {AdeH^+^···AdeH^+^}_∞_. In-plane 2D *XY* magnetism is characterized by a *T*_c_ close to 33 K, *H*_c,in-plane_ = 60 Oe, and *H*_c,out-of-plane_ = 750 Oe, high values of in-plane γ critical exponents (γ_b_ = 2.34(6) for *H*||*b* and γ_c_ = 2.16(5) for *H*||*c*), and a Berezinskii–Kosterlitz–Thouless (BKT) topological phase transition, deduced from crystal-orientation-dependent scaling analysis. The obtained values of in-plane ν critical exponents, ν_*b*_ = 0.48(5) for *H*||*b* and ν_*c*_ = 0.49(3) for *H*||*c*, confirm the BKT transition (ν_BKT_ = 0.5). Full-range angle-resolved monocrystalline magnetic measurements supported by dedicated calculations indicated the occurrence of nonlinear susceptibility performance within the easy plane in a magnetically ordered state. We refer the occurrence of this phenomenon to spontaneous resolution in the *C*2 space group, a tandem not observed in studies on previous analogues and rarely reported in the field of molecular materials. The above *magneto-supramolecular strategy* may provide a novel means for the design of 2D molecular magnetic networks and help to uncover the inherent phenomena.

## Abstract 2

The interbilayer distance is engineered by the complex synthons between adeninium cations and a rare four-fold symmetry of magnetization is present operating in the (AdeH){Cu[W(CN)_8_]}·2H_2_O **1** magnet in the long-range magnetic ordering state.

## Introduction

Layered inorganic materials, e.g., magnetic, conducting, semiconducting, and so on, has gained interest owing to its high potential in construction of spintronic composites, e.g., multilayered spin valves exploiting giant magneto-resistance. In reference to and parallel to the first-choice components toward practical applications (graphene, metals, metalloids, their oxides or chalcogenides, and alloys), modern molecular and coordination chemistry quickly created a parallel extensive research field.^1,2^ Very recently, various aspects of formation, flexibility, properties and application perspectives of 2D coordination polymers were very thoroughly reviewed by Vittal and co-workers.^3^ Indeed, offering optical transparency, soft matter character, and generally environmentally friendly mild synthesis conditions, layered coordination networks opened a relatively easy access path toward a combination of magnetic and optical features, together with their external control.^4^ Some layered coordination motifs appear systematically under particular synthesis conditions, and their functionality can be tuned using the variety of accompanying species (anions, cations, and neutral species). Thus, from the viewpoint of crystal engineering, they could be considered secondary building units (SBUs), with structural and functional properties tunable by crystallizing or cocrystallizing agents. Such an approach has been widely employed to achieve a diversity of spin-crossover (SCO) transitions in Hofmann-type M[M′(CN)_4_] (M = Fe^II^; M′ = Ni^II^, Pd^II^, and Pt^II^) clathrates,^5−11^ implementation of various additional functions into layered oxalates {M[M′(C_2_O_4_)_3_]}^0/–^^12−14^ or anilates {M[M′(C_6_O_4_X_2_)_3_]}^−^^15−18^ (M, M′ = divalent or trivalent 3d metal ions), engineering of interlayer separation and long-range magnetic ordering (LRMO) in hybrid layered hydroxides,^19,20^ tuning of LRMO in thiocyanato-bridged solid solutions [Co_*x*_Ni_1–*x*_(NCS)_2_(ligand)_2_]_*n*_,^21^ or the very recent electrochemical modification of interlayer nanospace in newly established Cr(pyrazine) family.^22,23^ A significant contribution to the field is provided by cyanide-bridged networks with recurrent modular 2D bimetallic backbones MM′·*ligand*·*guest* involving exchangeable [M′(CN)_8_]^3–/4–^ (M′ = Mo, W, Re, and Nb) and di- or trivalent 3d metal ions (Mn, Fe, Co, Ni, and Cu), or trivalent 4f ions, M.^24^ Various combinations of components did operate within a limited number of topologies to shape LRMO and its anisotropy,^25−27^ which was frequently accompanied by externally controlled change in solvate/guest composition,^28,29^ charge transfer and spin transition phenomena,^30−34^ and charge carrier injection/extraction^35,36^ or flow^37,38^ phenomena.

**Figure 1:**
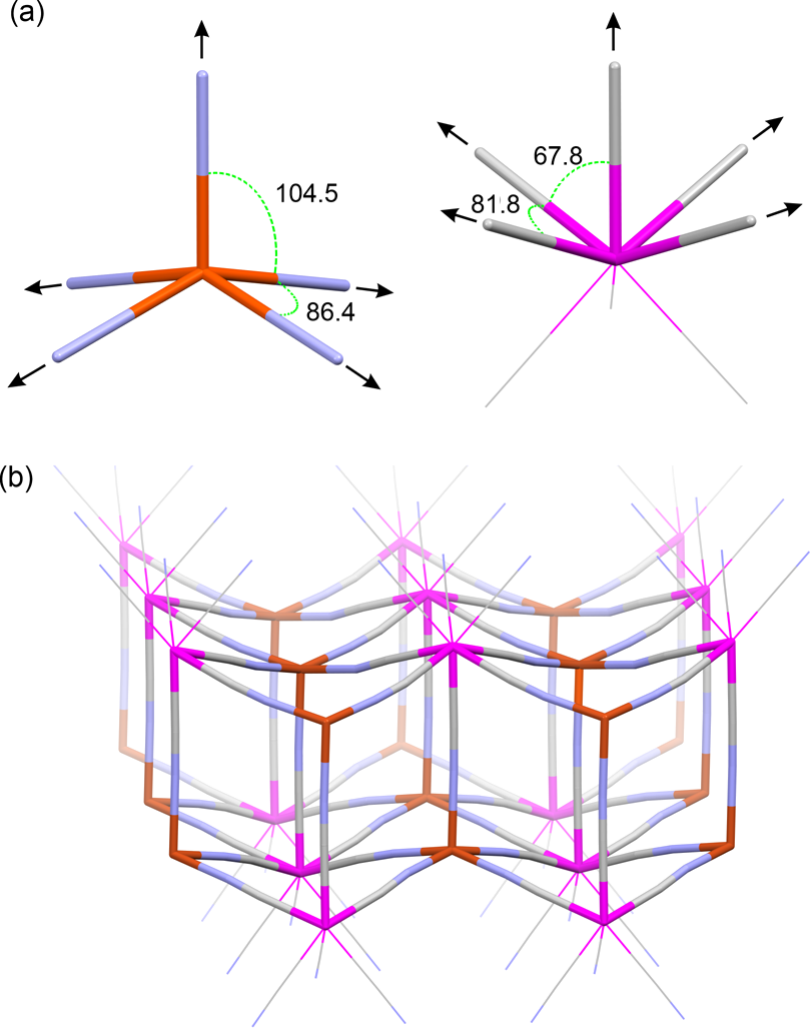
(a) The stereochemistry of the ideal square pyramid (SPY-5) polyhedron (left) almost ideally fits to the distribution of the relevant five out of eight cyanido ligands in a biaugmented trigonal prism polyhedron (representative also for bicapped trigonal prism) (BTRP-8) (right). (b) This allows to us construct the bilayered skeleton in a reproducible manner.^39−42^ Some octahedral complexes might adopt this topology with an appropriate fine distortion as well.^43−46^ Color and style scheme: pink, W; brick, Cu; pale gray; N, pale blue; sticks: bridging, CN^–^; wireframe, terminal CN^–^. The arrows show a directionality of cyanido-bridges formation. The angles L–M–L refer to the ideal polyhedra provided within the SHAPE package.^47^

Among the above compounds, the anionic {M^II^[M′^V^(CN)_8_]}^−^ (M = Cu and Mn; M′ = Mo and W) bilayers constitute a good example of a systematically recurrent molecular module. The double-decker topological Prussian Blue Analogue (PBA) fragment is achieved due to a stereochemical matching between the square pyramidal (SPY-5) shape of Cu^II^ moiety or octahedral shape of Mn^II^ moiety, and the bicapped trigonal prism (BTRP-8) [W^V^(μ-CN)_5_(CN)_3_] unit (Figure 1).^39−47^ Such double-decker fragments were previously put together by various cationic species: tetrenH_5_^5+^ (CuM′),^39^ dienH_3_^3+^ (CuM′),^40^ Cs^+^ (CuW, CuMo),^41^ guanidinium (guaH^+^) (CuM′),^42^ single Cu^2+^ (CuMo,CuW),^43,44^ and Mn^2+^ (MnMo)^45^ complexes or polymeric 1D {Cu(μ-pyz)}^2+^ chains (CuM′).^46^ The average interlayer distances varied between ca. 8 and 11 Å, which resulted in LRMO below *T*_c_ between 27 and 43 K and coercive field *H*_c_ between ca. 2500 Oe and ca. 80 Oe, both parameters decreasing with the increasing interlayer distance in the case of CuM′. The in-plane *XY* magnetism of a single CuW bilayer was recently exploited in the studies of inverse magnetocaloric effect (MCE) and rotational MCE (RMCE),^48^ whereas the MnMo bilayers served as platforms for reversible cation (Li^+^,Na^+^)-electron pairs injections/extractions processes, essential from the viewpoint of the fabrication of lithium batteries.^45^

In this work, we make a step toward crystal engineering of coordination layered magnets using topologically advanced components. This inspiration came from the field of biochemistry: the purine- and pyrimidine-type nucleic bases offer the unique side double or triple hydrogen bond patterns owing to the specific distribution of N-heteroatoms and N–H, C=O, and NH_2_ functions.^49−52^ Considering the above, and driven also by simple curiosity, we carried out self-assembly tests toward the formation of new solid phases involving {Cu^II^[M^V^(CN)_8_]}^−^, using acidic aqueous solutions as a medium. As a result, we present the crystal structure and complete angle-resolved magnetic studies of (AdeH){Cu^II^[W^V^(CN)_8_]}·2H_2_O (**1**) (AdeH^+^, adeninium cations). The new solution provides significantly enlarged interbilayer separation, possible chirality, and the occurrence of nonlinear susceptibility performance in the magnetically ordered state; the last envisaged by full-range angle-resolved monocrystalline magnetic measurements were supported by dedicated calculations.

## Experimental Section

Precursors were purchased from commercial sources (Sigma-Aldrich, Idalia, Alfa Aesar) or synthesized using literature methods.^53^ SC XRD (model **1**) data were collected on BESSY II synchrotron BL14–3 beamline (Helmholtz Zentrum Berlin, Bessy II)^54^ and processed with *xdsapp*(55,56) and CrysAlis Pro^57^ (absorption correction), SHELXS and SHELX 2018/1^58^ (solution and refinement), and WinGx/ROTAX^59^ (solution of twinning) softwares. PXRD data (model **1p**) were processed using EXPO2014^60^ (indexing and preliminary structure model), FOX^61^ (structure determination and optimization), and JANA2006^62^ (refinement) software, considering the previous bilayer structural model^39^ and CSD entries containing AdeH^+^ cations.^63^ All structural figures were prepared in Mercury.^64^ Continuous shape measure analysis of coordination spheres was carried out using SHAPE 2.1.^47^ The crystal structures are deposited in the CCDC database, with the deposition numbers 2058785 (model **1**) and 2058786 (model **1p**). The detailed description of synthesis procedures, crystal data collection, crystal structure determination and refinement, as well as physical methods are available in Figures S1–S3, and S4a and Tables S1–S3.

## Results and Discussion

### AdeH^+^ as a Supramolecular Tecton

**Figure 2:**
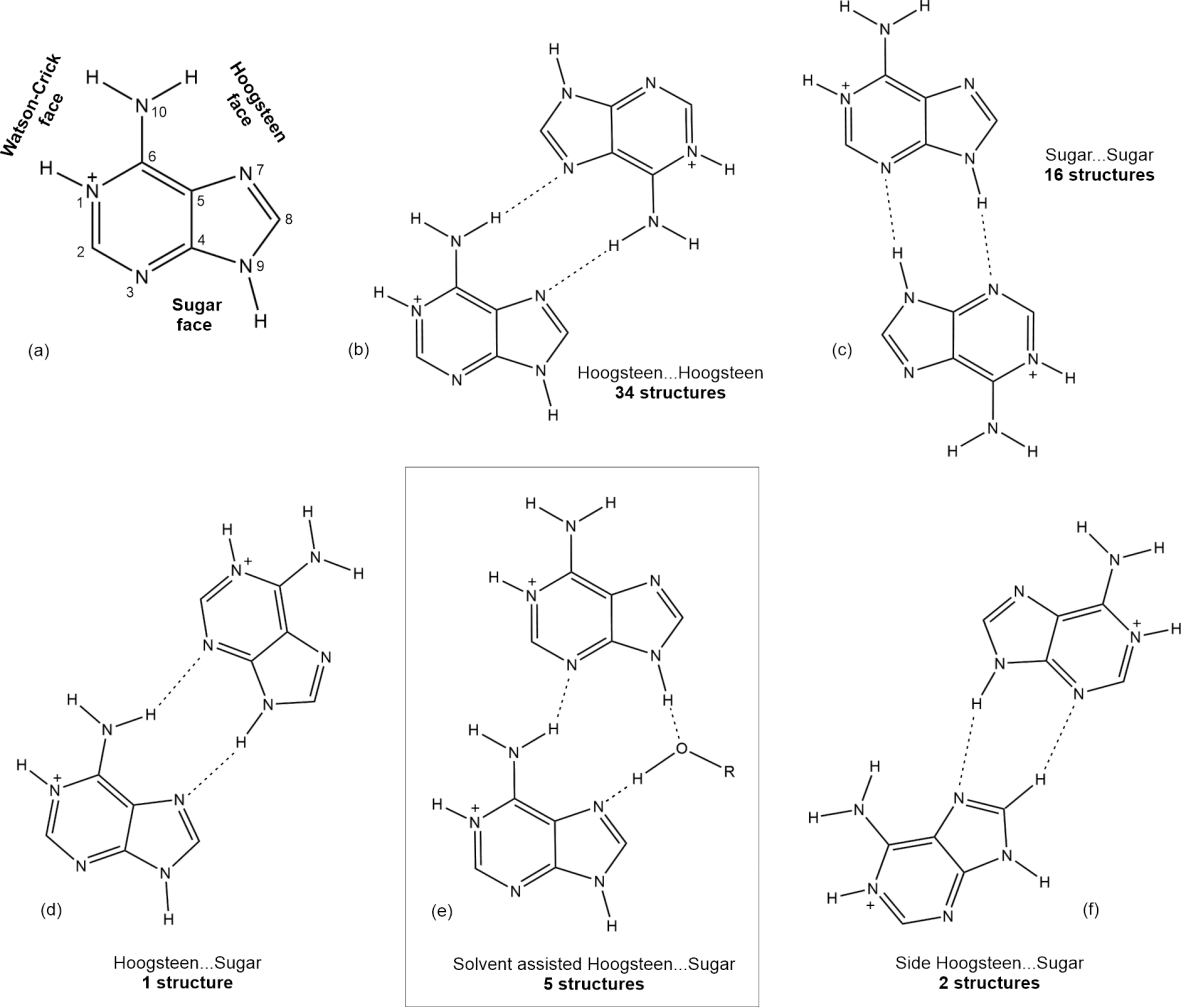
(a) Location of the Hoogsteen face, the sugar face and the Watson–Crick face in AdeH^+^. (b–f) Dimeric in-plane synthons between AdeH^+^ cations based on CSD data (2020.1) together with their counts, including the synthon observed in **1** (e). Protonation at the N^1^ atom was assumed on the basis of literature data.^65−70^ The detailed constrains and the full list of structures (CSD refcodes) are presented in Figure S5 and Table S1.

**Figure 3:**
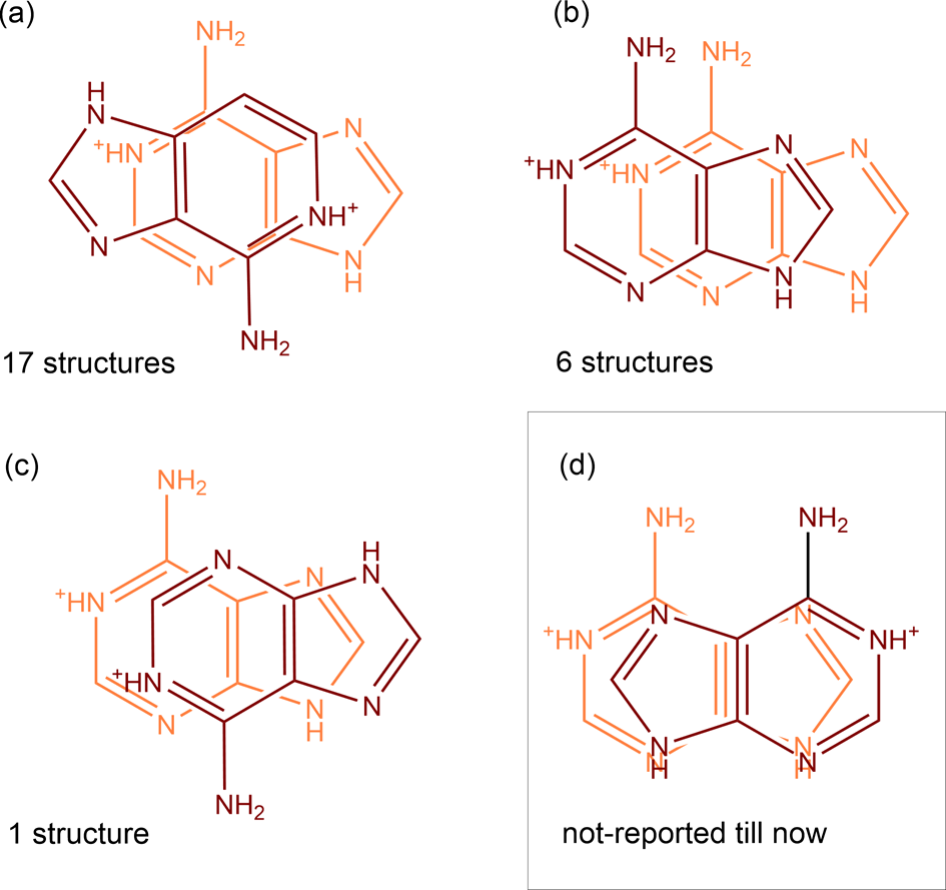
Dimeric parallel or offset parallel synthons between the adeninium cations based on CSD data (2020.1) together with their counts (a–c). The synthon observed in **1** was not observed in database until now (d). The distance between the neighboring AdeH^+^ planes is not higher than 3.7 Å. The detailed constraints and a full list of the related CSD reference codes are presented in Figure S6 and Table S2.

AdeH^+^ cations form parallel planar or close-to-planar side {AdeH^+^···AdeH^+^} synthons (Figures 2 and S5 and Table S1) exploiting interactions between their distinguished regions of the Hoogsteen face (HN^10^–C^6^–C^5^–N^7^), and the sugar face (N^3^–C^4^–N^9^H) (for an example description and location of protons, see refs (65−70); Figure 2a). The full record of crystal structures detected includes: 34 cases with the Hoogsteen···Hoogsteen synthon (Figure 2b), 16 cases with the sugar···sugar synthon (Figure 2c), and finally 8 cases with various Hoogsteen···sugar synthons, counting 1 structure with a direct contact (Figure 2d), 5 structures with solvent-assisted contacts, (Figure 2e), and 2 structures exhibiting a contact between the sugar face and the N^7^C^8^H side fragment of the Hoogsteen face (Figure 2f). The third region, the protonated Watson–Crick face (HN^10^–C^6^–N^1^–H) for the obvious reason is not involved in the {AdeH^+^···AdeH^+^} synthons; instead, it usually binds nucleophilic entities (mostly anions). The aforementioned planar synthons are frequently accompanied by the parallel or offset parallel π–π interactions with interplanar distance down to ca. 3.1 Å (Figures 3 and S6, Table S2). Such a diversity of intermolecular {AdeH^+^···AdeH^+^} synthons and their tendency to form stacked arrangement offers the perspective of using the related *infinite cationic blocklike synthons* for molecular crystal engineering.

### Structural Studies

The crystal structure of **1** was determined after a thorough analysis of SC XRD data obtained using synchrotron radiation (model **1**). The crystal data and structure refinement parameters are presented in Table S3. **1** crystallizes within the monoclinic *C*2 space group (*a* = 41.3174(12), *b* = 7.0727(3), *c* = 7.3180(2) Å, β = 93.119(3)°, and *V* = 2135 Å^3^).

**Figure 4:**
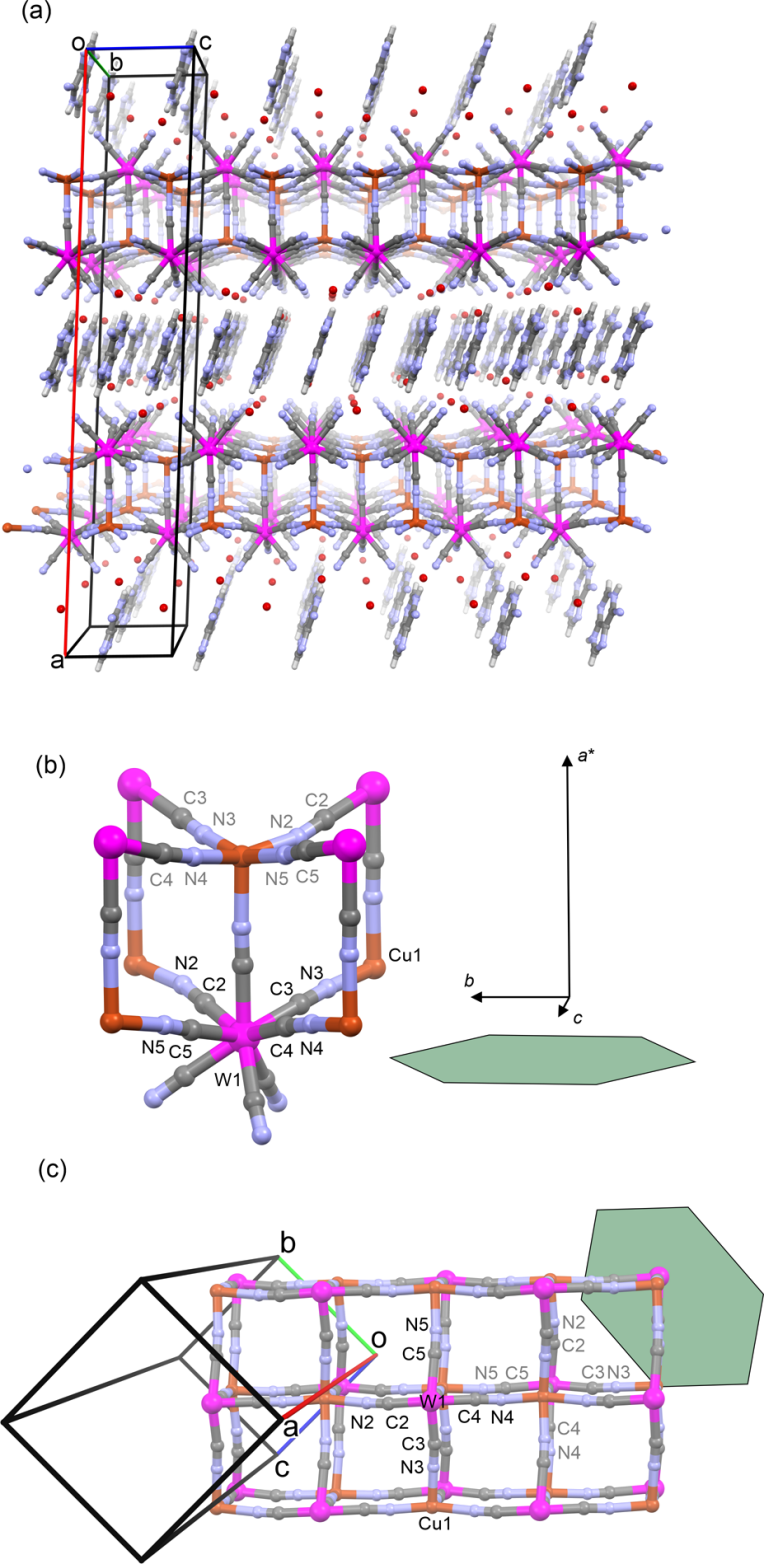
Crystal structure of **1**. (a) 3D architecture showing anionic coordination double layers {Cu^II^[M^V^(CN)_8_]}^−^ separated by AdeH^+^ cations (see also Figures 2e and 5). (b, c) Fragments of coordination backbone showing the arrangement of cyanido-bridged linkages along the bilayer, crystallographic directions, and atom numbering according to the SC XRD model **1**. The hexagonal green shape fairly illustrates the crystal habit and its orientation in respect to the crystallographic directions and cyanido-bridged linkages. Colors: W, pink; Cu, brick; C, gray; N, pale blue; O, red.

Detailed information on the intralayer distances, bond lengths, angles, and types of coordination polyhedra are presented in the upper section of Table 1 and in Figure S7 and Tables S4 and S5. The topological motif of the 2D cyanido-bridged network {Cu^II^[W^V^(CN)_8_]^−^}_*n*_ with four equatorial and one axial cyanido-bridged linkage was reproduced (Figure 4a). The Cu_eq_···W distances (5.23–5.26 Å) are significantly shorter compared to the Cu_ax_···W distance (5.44 Å), in a good agreement with the data shown previously.^39−42^ The underlying bond lengths Cu–N_eq_ (1.92–2.04 Å), Cu–N_ax_ (2.129 Å), W–C (2.09–2.19 Å), and C–N (1.09–1.20 Å), and the relevant angles W–C–N (172.8–176.3°), Cu–N–C_eq_ (167.4–175.3°) and Cu–N–C_ax_ (177.4°) are in line with the recently investigated structural models. The directions of equatorial cyanido-bridges are oriented diagonally with respect to the crystallographic directions *b* and *c*, whereas the axial bridges are parallel to the direction a*. The {···N3–Cu–N5C5–W–C3···}_∞_ and {···N2–Cu–N4C4–W–C2···}_∞_ linear chains forming square grid arrangement in the bottom deck of the bilayer are also perpendicular to those in the top deck (Figure 4b,c), and this feature is repeated in each bilayer.

**Table 1:** Most Important Intermetallic, Intralayer, and Interlayer Distances in Structural Model **1** and Their Comparison with the Previously Reported Compounds

	**1**	tetrenH_5_^5+^ CuW^39^	dienH_3_^3+^ CuW^40^	Cs^+^ CuW^41^	guaH^+^ CuW^42^
(a) Average Intralayer Distances (Å)					
Cu_eq_–W^a^	5.24	5.25	5.24	5.25	5.24
Cu_ax_–W	5.44	5.44	5.44	5.44	5.43
Cu–Cu^b^	6.89	6.89	6.88	6.91	6.89
W–W^b^	7.81	7.81	7.81	7.80	7.81
(b) Average Interlayer Distances (Å)					
Cu···Cu^c^	**16.89**	12.40	12.21	11.40	12.19
Cu···W^c^	**15.64**	11.24	11.06	10.16	10.96
W···W^c^	**14.44**	9.97	9.80	9.12	9.84
W_planes_^d^	**13.92**	9.34	9.15	8.20	8.94
Cu_planes_^d^	**16.46**	11.89	11.70	10.67	11.47
(c) Magnetic Properties					
*T*_c_/K	32	34	34	32.8	30
*H*_c_/Oe	60	80	225	300	90

a The average distance between the central atom and 4 adjacent atoms

b The average distance between the central atom and 8 adjacent atoms.

c The average distance between the central atom of one bilayer and the nearest atoms from the neighboring layer.

d The distance between two planes determined by positions of corresponding atoms from the first layer and from the neighboring layer.

**Figure 5:**
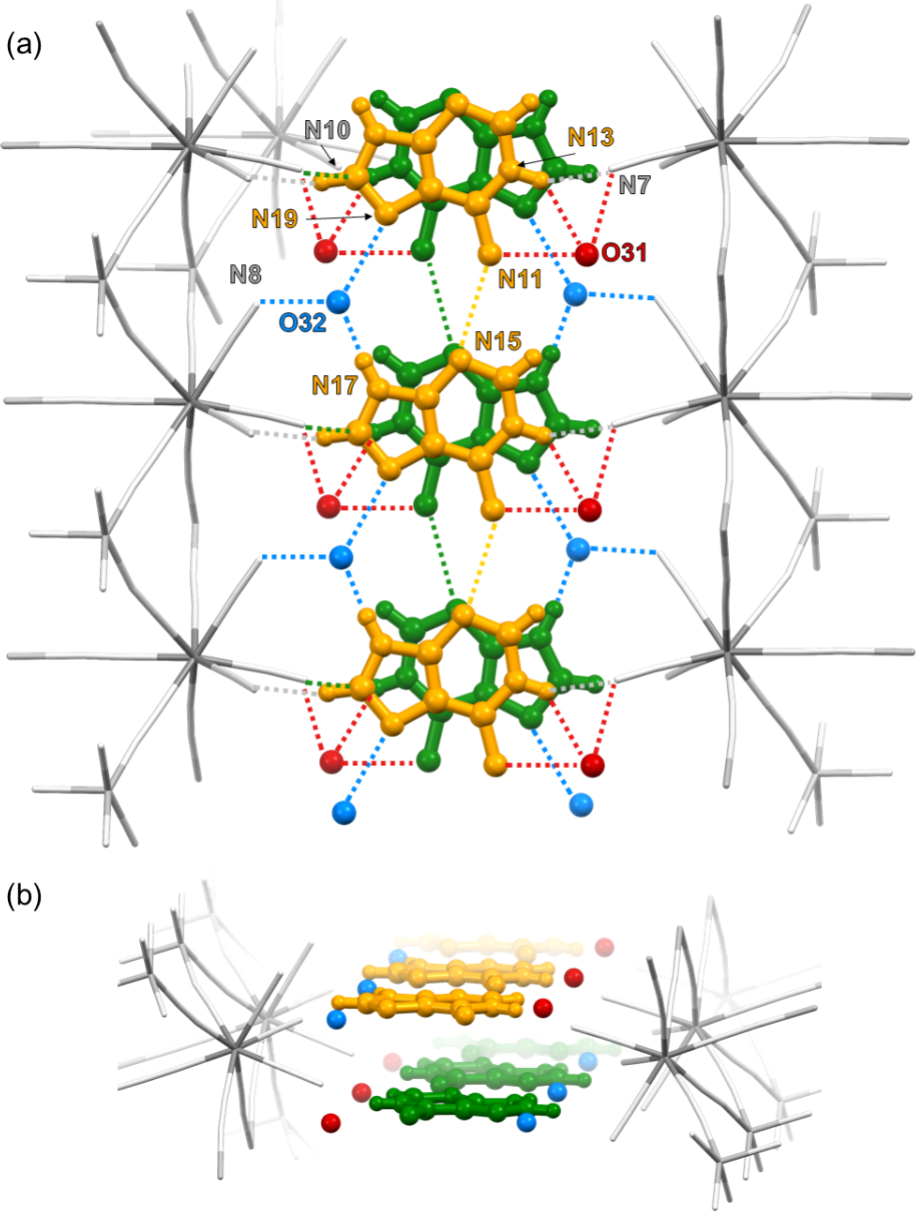
Illustration of hydrogen bonds and short contacts in **1**. Color code: green and orange, AdeH^+^ cations in two adjacent planes; red, oxygen atom of H_2_O(31) molecules located slightly out of the AdeH^+^ plane; blue, oxygen atom of H_2_O(32) molecules located in the AdeH^+^ plane; gray scale, coordination skeleton of the Cu^II^W^V^ bilayer. For details see Table 2. Compare with Figure 2e.

The illustrative data on interlayer separation are shown in the bottom section of Table 1. All examined parameters, the closest Cu···Cu, Cu···W, and W···W separations, together with the closest distances between the planes formed by Cu atoms (or W atoms) belonging to the neighboring layers (W_planes_ and Cu_planes_, respectively), indicate a significant increase of the interlayer separation of ca. 50% compared to the former analogues in this family. This was achieved owing to the specific molecular 2D arrangement of the adeninium AdeH^+^ cations combining the in-plane contacts and stacking contacts, as illustrated in Figure 5 and Table 2. The in-plane AdeH^+^···AdeH^+^ synthons are arranged into infinite {AdeH^+^}_*n*_ ribbons *via* direct double-strand contacts between the Hoogsteen face (HN^10^–C^6^–C^5^–N^7^) and the Sugar face (N^3^–C^4^–N^9^H), here realized by the N15···H–N11 contact (*d*_NN_ = 3.06 Å) (N^3^···H–N^10^) accompanied by the N19···H–O32···H–N17 contact (*d*_NO_ = 2.80 and 2.68 Å) (N^7^···H–O···H–N^9^) (compare Figure 2a,e). The O32 atom of the water molecule forms also a contact with the N8C8 terminal cyanide, most probably acting as the hydrogen donor. The supramolecular architecture is completed with a contact between the protonated Watson–Crick face (HN^10^–C^6^–N^1^–H) and O31 water molecule (N11···O31 = 2.70 Å), which is additionally bound to the N7C7 cyanide at the other side of {AdeH^+^}_*n*_ ribbon (O31···N7 = 2.62 Å). The neighboring ribbons are parallel to each other and form infinite stacks within 2D interlayer space, with the interplane distance of ca. 3.4 Å. Within this arrangement, the sugar faces are projected onto each other at the one side of the stack, and the Hoogsteen faces are projected onto each other at the second side. However, the protonated Watson–Crick faces are exposed remotely outside the center of AdeH^+^. It is important to note that the in-plane solvent-assisted Hoogsteen···sugar synthons observed in **1** were realized previously in crystal structures of 5 literature compounds (CSD crystal structure refcodes EGOWIG, LOLDEW, LOLDIA, UWAMEM, and UWAMEM1; Figure 2e) exclusively involving monoprotonated AdeH^+^ synthons. This significantly supports a reliability of our observation. The C–C and C–N distances in AdeH^+^ cations are close to those found in the reference literature data (Figures S8, and S9, Table S6).

**Table 2:** H-Bonds Donor···Acceptor Distances in **1**^a^

D	A	*d*_DA_	d_DA_ – ∑VdW	angle D–H···A[deg]
N7	N13	2.87	–0.24	150
N10	N13	3.07	–0.03	105
N7	O31	2.62	–0.45	
N8	O32	2.53	–0.54	
N15	N11	3.06	–0.04	
O31	N11	2.70	–0.37	
N19	O32	2.80	–0.274	
O32	N17	2.68	–0.394	175

a The role of donor (D) and acceptor (A) were determined intuitively, where required.

Analysis of the PXRD data (see model **1p**, Figures S10 and S11, Tables S3, S7, and S8) showed the presence of {Cu^II^[M^V^(CN)_8_]}^−^ bilayers, AdeH^+^ cations, and crystallization H_2_O molecules in **1**. It also confirmed a crucial role of the latter components in shaping of the interbilayer separation in **1** (for more detail, see the Supporting Information).

Chirality that could occur in the space group *C*2 is canceled by a 4-component twinning involving inversion operation necessary to model the crystal structure in SC XRD data analysis; however, the specific bilayer arrangement observed for the single primary grain will have substantial impact on magnetic properties. The twinning is dominated by the component obtained through the inversion operation of the original grain structure (47.5%) and completed by other minor components issued by the *C*_2_ rotation with respect to the [100] direction (2.27%, see the description of TWIN command in the CCDC file), and by a combination of this rotation with the inversion operation (3.26%). Considering the fact that magnetization is a pseudovector (it does not change under the inversion operation), such twinning composition should not influence the magnetic properties.

### Magnetic Properties

**Figure 6:**
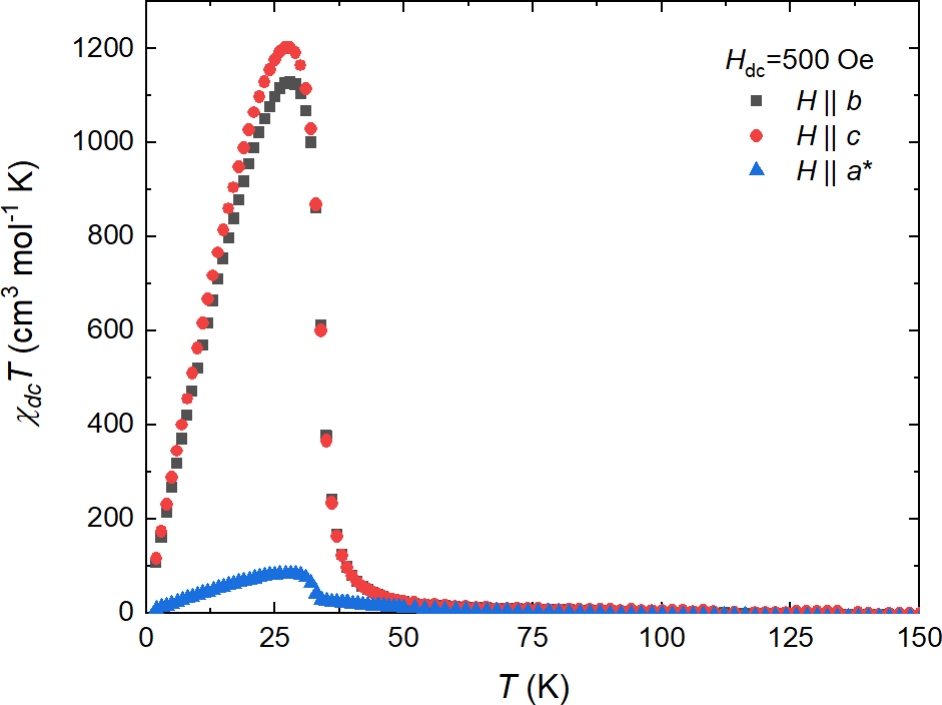
Temperature dependence of χ*T* for **1** as measured at 500 Oe for *H*||*b* (black squares), *H*||*c* (red circles), and *H*||a* (blue triangles).

**Figure 7:**
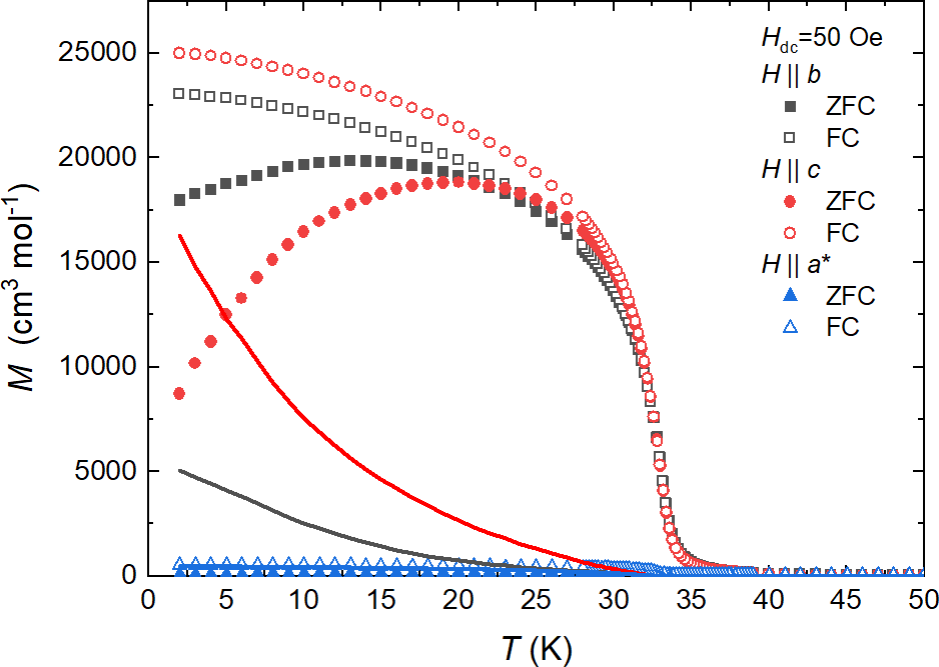
Zero-field-cooled (full symbols) and field-cooled (empty symbols) magnetization for *H*_dc_ = 50 Oe as a function of temperature for *H*||*b* (black squares), *H*||*c* (red circles), and *H*||a* (blue triangles). The solid lines show the difference between the FC and ZFC data.

Magnetic measurements for a batch of single crystals were carried out along three orthogonal directions, *H*||a*, *H*||*b*, and *H*||*c*, indicated by indexing procedure and in accordance with solution model **1** (Figure S12). Figure 6 shows the temperature dependences of χ*T* products with an applied field of 500 Oe. In the case of *H*||*b* and *H*||*c* orientations, a sharp increase of χ*T* values start below 40 K, whereas for the direction perpendicular to the layer (*H*||a*), a noticeable growth can be observed below 35 K. For all orientations, the maximum values were recorded at *T*_peak_ = 28.0 K, reaching 1130 cm^3^ mol^–1^ K for *H*||*b*, 1202 cm^3^ mol^–1^ K for *H*||*c*, and 84 cm^3^ mol^–1^ K for *H*||a*. It is clear that the magnetization prefers to align within the double-layer (within the *bc* plane), while the perpendicular direction, with the response more than 1 order of magnitude smaller, is the hard axis. However, much smaller but still noticeable differences can be observed between the two in-plane orientations. These dissimilarities are more evident in low field (50 Oe) ZFC/FC measurements (Figure 7 and S13), which show stronger difference between field-cooled and zero-field-cooled curves for *H*||*c* orientation than for those *H*||*b*. This suggests a more complex anisotropy than the simple easy plane type. A notable divergence between ZFC and FC curves starts for all orientations at a similar temperature of about 32.6 K (Figure S13), while the *T*_c_ temperature, determined from the first derivative of ZFC susceptibility (Figure S14), is slightly higher: *T*_C_ = 32.8(2) K for *H*||a*, *T*_c_ = 33.2(2) K for *H*||*b*, and *T*_c_ = 33.0(2) K for *H*||*c*.

**Figure 8:**
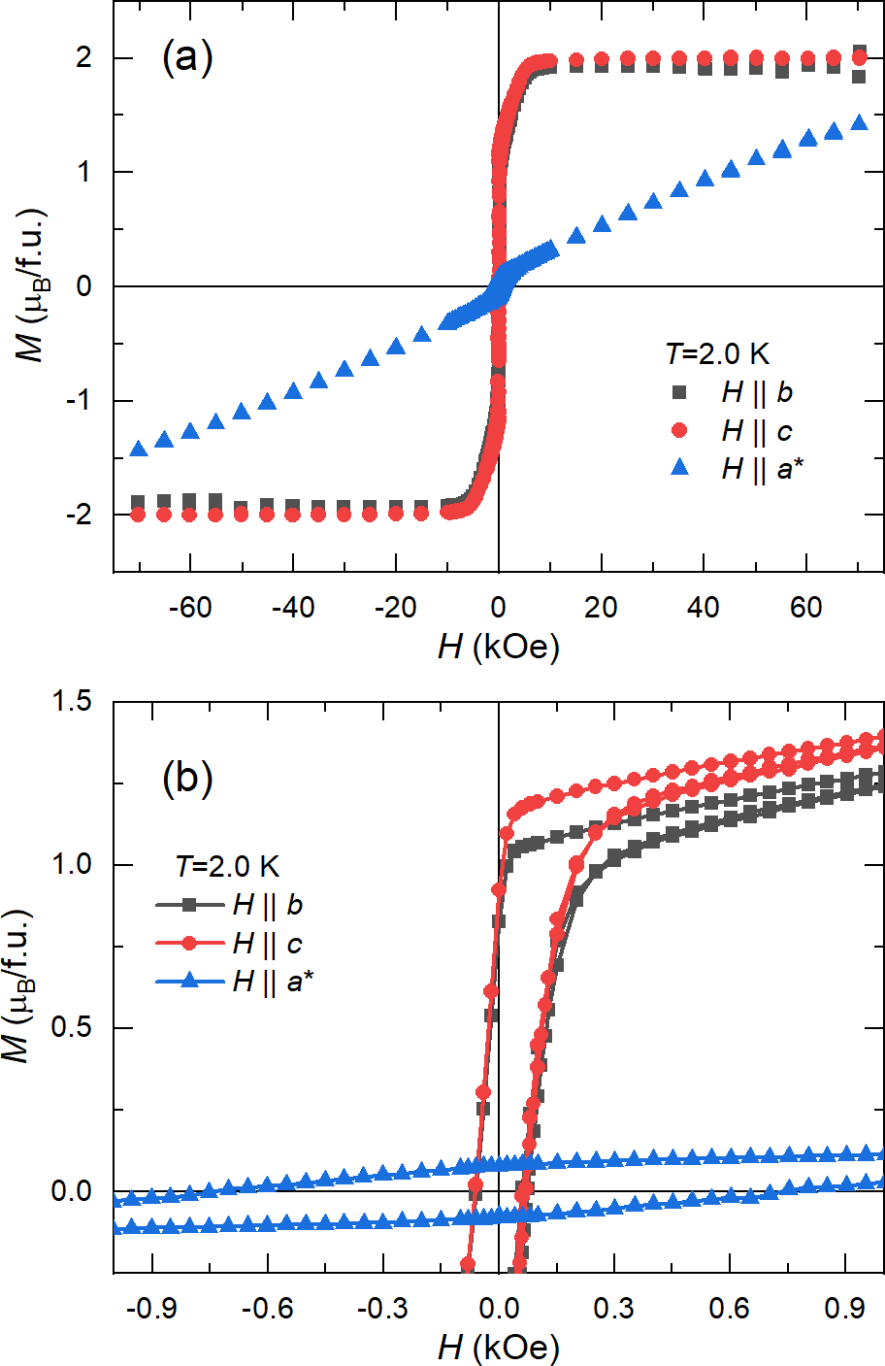
Isothermal magnetization at 2.0 K for *H*||*b* (black squares), *H*||*c* (red circles), and *H*||a* (blue triangles) orientations.

Isothermal magnetization measurements at 2.0 K (Figure 8) indicate that **1** saturates for *H*||*b* and *H*||*c* for fields above 10 kOe at 2.0 μ_B_/f.u., which agrees with the expected value for the parallel alignment of the Cu^II^ (*S*_Cu_ = 1/2, *g*_Cu_ = 2.0) and W^V^ (*S*_W_ = 1/2, *g*_W_ = 2.0) magnetic moments per {Cu^II^[W^V^(CN)_8_]}^−^ unit. In contrast, the applied field in the perpendicular direction, even at 70 kOe, is too weak to reach saturation, which points to a significant magnetic anisotropy. Within a double-layer, **1** displays a rather soft magnetic behavior with the small coercive field of 60 Oe, whereas coercivity for *H*||a* is 1 order of magnitude higher reaching 750 Oe.

Both the isothermal magnetization and the temperature dependence of magnetic susceptibility points to a ferromagnetic behavior with significant intramolecular interactions within the *bc* plane. Since the interbilayer separation is substantial (about 14.5 Å) and there is no direct linkage between the planes, the most obvious candidate for interbilayer interaction is the dipole–dipole coupling.

### Angle-Resolved Susceptibilities

**Figure 9:**
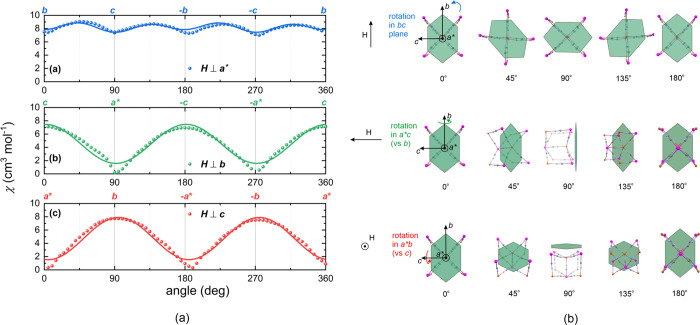
Angle-resolved monocrystalline magnetic measurements: (a) magnetic susceptibility in three independent crystallographic planes at 2 K (solid lines show the best fit of the nonlinear model); (b) the respective orientations of the statistic rotated crystals in the SQUID cavity in respect of the direction of the external magnetic field. Curved arrows show the rotation of the crystal. The hexagonal crystal’s shape in the figure fairly illustrates the real crystal’s shape (compare Figure S12).

The magnetic susceptibility was measured within three independent planes, i.e., the plane perpendicular to the a* crystallographic axis, the plane perpendicular to the *b* crystallographic axis, and the plane perpendicular to the *c* crystallographic axis, with the angle step of 5° in the applied field of 1 kOe at 2 K, using a Quantum Design Horizontal Sample Rotator and Quantum Design MPMS XL magnetometer. The raw data were corrected for diamagnetic contribution using the orientation averaged value of χ_0_ ≈ −0.015 cm^3^ mol^–1^ per formula unit (see Figures S15 and S16). The measurement results shown in Figure 9 confirm that the a* crystallographic axis corresponds to the hard magnetization direction, while the *bc* crystallographic plane constitutes the easy magnetization plane.

**Figure 10:**
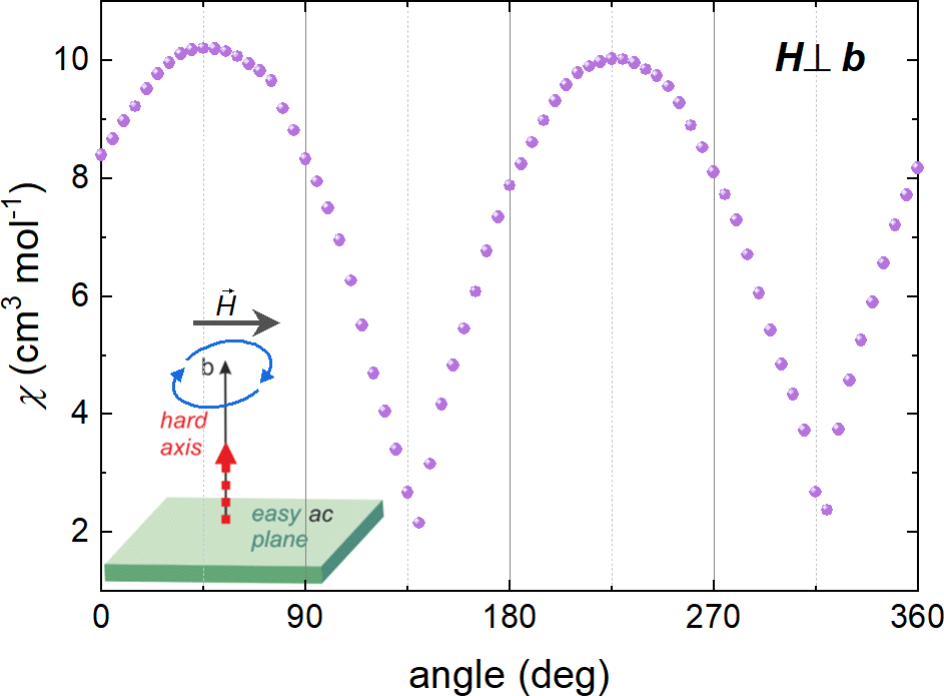
Angle-dependent dc susceptibility of (tetrenH_5_)_0.8_{Cu^II^[W^V^(CN)_8_]_4_}·7.2H_2_O}_*n*_ (**2**)^48^ at 2.0 K and in 1 kOe applied field.

There is, however, one additional striking feature of angle-resolved measurements: A rotation within the easy plane (*bc*) indicates the presence of a 4-fold axis, which contrasts with 2-fold symmetry indicated by rotations within the planes parallel to the hard axis (a*b and ca*). This feature cannot be explained within the linear paradigm, where only the second-rank susceptibility tensor χ_αβ_ is employed. To check if a 4-fold axis is a common feature of the Cu^II^–W^V^ double-decker topological Prussian blue analogues family, angle-resolved measurement of (tetrenH_5_)_0.8_{Cu^II^[W^V^(CN)_8_]_4_}·7.2H_2_O}_*n*_^48,71^ (**2**) (Figure 10) was carried out within the *ac* easy plane, with respect to the direction *b*, and perpendicular to crystal plane. Although the crystal structures of **1** and **2** are very similar to each other, the latter compound shows only a 2-fold axis. It could be that in the case of **2** the applied field is too weak to reveal the nonlinear behavior; however, this issue will be analyzed in more detail in further studies. In what follows, we will show within a simplified model that the 4-fold symmetry in **1** can be roughly reproduced assuming a higher-order contribution to the susceptibility which is nonlinear in the applied field.

Let us expand the magnetization pseudovector in a series of the applied magnetic field:
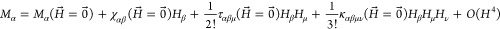
1where the Einstein summation rule over repeated indices has been assumed. The relation  eliminates all even field terms in the expansion in eq 1, so that  and  for all α, β, μ ∈ {a*, *b*, *c*}. Thus, the measured susceptibility  corresponding to the applied field vector  reads
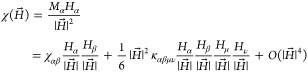
2where
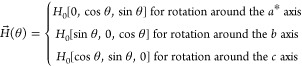
3with *H*_0_ = 1 kOe and the rotation angle θ ∈ [0, 360°). We assume that the susceptibility linear tensor χ_αβ_ is symmetric, i.e., χ_αβ_ = χ_βα_, while the only nonvanishing components of the nonlinear tensor κ_αβμν_ are those with indices *bbcc* and all different permutations thereof. Moreover, we assume that they are all equal as being of the same symmetry, i.e., κ_*bbcc*_= κ_*bccb*_= κ_*ccbb*_= κ_*cbbc*_= κ_*bcbc*_= κ_*cbcb*_ ≠ 0. Then, a straightforward calculation using eqs 2 and 3 yields
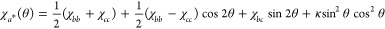
4
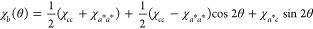
5
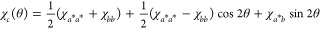
6where κ = *H*_0_^2^κ_*bbcc*_. Equations 4–6 have been simultaneously fitted to the experimental angle-resolved susceptibility data using the following test function (agreement quotient)
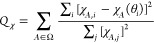
7where Ω = {a*, *b*, *c*} is the set of the rotation axes. The best fit yielded *Q*_χ_ = 2.02 × 10^–2^ and the set of parameter values listed in Table 3. The solid lines in Figure 8 show the best-fit curves.

**Table 3:** Parameter Values Corresponding to the Best Fit of the Nonlinear Model to the Angle-Resolved Susceptibility Data

parameter	χ_a*a*_ [cm^3^ mol^–1^]	χ_*bb*_ [cm^3^ mol^–1^]	χ_*cc*_ [cm^3^ mol^–1^]	χ_a*b_ [cm^3^ mol^–1^]	χ_*bc*_ [cm^3^ mol^–1^]	χ_ca*_[cm^3^ mol^–1^]	κ [cm^3^ mol^–1^]
parameter value	1.59	7.85	7.45	–0.565	0.102	0.166	4.38
standard relative error [%]	4.0	0.89	0.92	13	73	44	10

One can see that the agreement with the experimental data is satisfactory but by no means perfect. The main feature of the data, i.e., the fact that rotations within the planes parallel to the hard axis (a*b and ca*, red and green, respectively, in Figure 9) reveal the presence of a 2-fold axis, while a rotation within the easy plane (*bc*, blue in Figure 9) indicates the presence of a 4-fold axis and is duly reproduced. However, the present model fails to reproduce the low-value kinks observed for rotations around the *b* axis (green in Figure 9) and *c* axis (red in Figure 9). This may be understandable due to the fact that the model in its present shape is crucially simplified, neglecting 75 out of 81 components of the nonlinear susceptibility tensor κ_αβμν_. However, the exceptionally large relative error for χ_*bc*_ together with its relatively small absolute value make it practically redundant, showing that the model may be even further simplified. It is worth noting that the component χ_*bc*_ would automatically vanish if one assumed that the system is composed of two sublattices transformed one into another by the 90° rotation around the hard axis (a* crystallographic axis). Then, the components κ_*bbcc*_= κ_*bccb*_= κ_*ccbb*_= κ_*cbbc*_= κ_*bcbc*_= κ_*cbcb*_ ≠ 0 of the nonlinear susceptibility tensor of the fourth rank give the first nonzero contribution in its place. The {···N3–Cu–N5C5–W-C3···}_∞_ and {···N2–Cu–N4C4–W-C2···}_∞_ linear chains forming square grid arrangement in the bottom deck of the bilayer are also perpendicular to those in the top deck and this feature is repeated in each bilayer.

**Figure 11:**
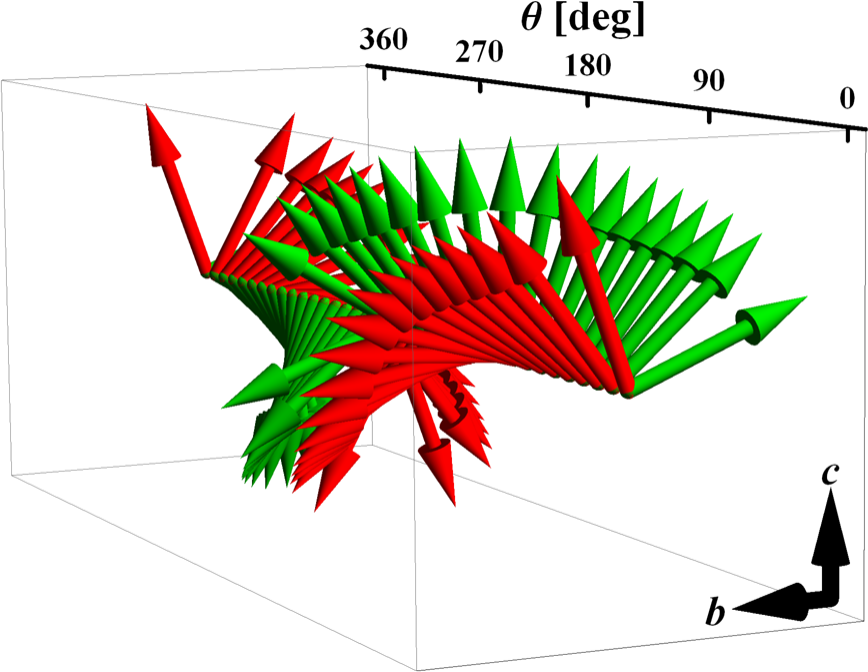
Rotation of eigenvectors of χ_a*αβ_ corresponding to the largest (red) and the second largest (green) eigenvalue as a function the orientation angle of the applied magnetic field .

**Figure 12:**
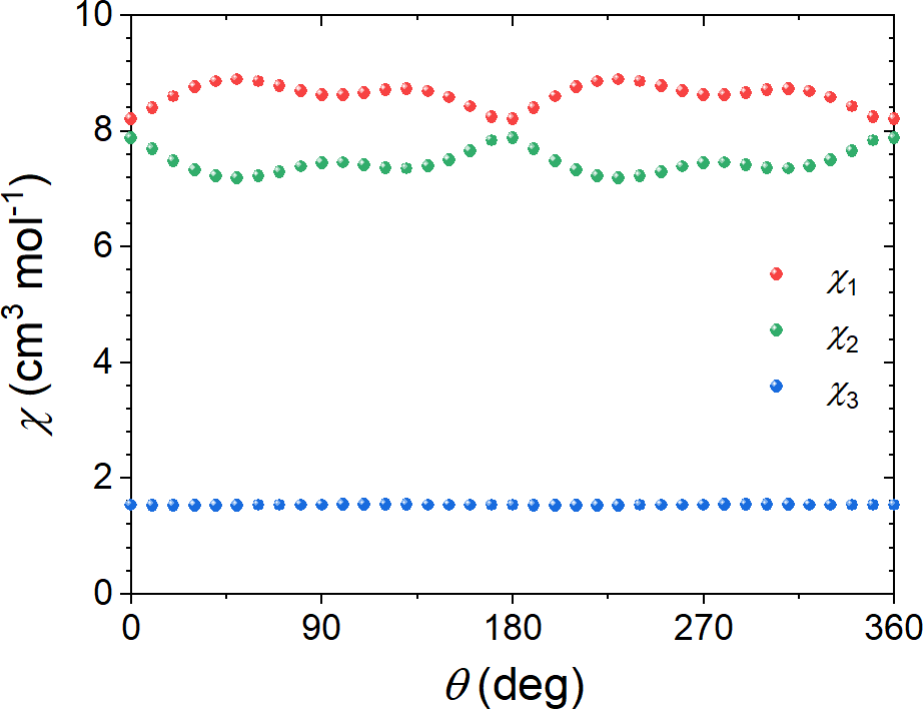
Dependence of the eigenvalues of χ_a*αβ_ on the orientation angle of the applied magnetic field .

In the above model, the nonzero components of the nonlinear susceptibility tensor κ_αβμν_ are crucial to reproduce the 4-fold symmetry axis for a rotation around the hard magnetization direction (a* crystallographic axis). At the same time, they give rise to an interesting feature of the total susceptibility tensor χ_a*αβ_ associated with that rotation:
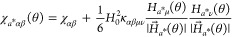
8where  is given by the first row in eq 3. It is apparent from eq 8 that due to the nonlinear susceptibility term the total susceptibility tensor χ_a*αβ_ becomes dependent on the orientation of the applied magnetic field expressed in terms of the rotation angle θ. Hence its eigenvalues and eigenvectors will also depend on the applied field orientation. Figures 11 and 12 show this dependence. It can be seen that the eigenvectors corresponding to the largest and the second largest eigenvalues lie in the *bc* easy plane and rotate in an anticlockwise sense around the a* axis with a rotation of the applied field . In this way, the direction of the external magnetic field coincides with the direction of the easy axis (red arrows in Figure 11) four times for angles θ roughly equal to 45, 135, 225, and 315°, which correspond to the local maxima of the largest eigenvalue (red symbols in Figure 12).

The quality of fit of the angle-resolved susceptibility data may be improved by adding to the present model other components of the four-rank tensor κ_αβμν_ as well as by extending the magnetization expansion in eq 1 to even higher orders. This is corroborated by the trigonometric polynomial approximation (see the Supporting Information).

### Scaling Analysis

**Figure 13:**
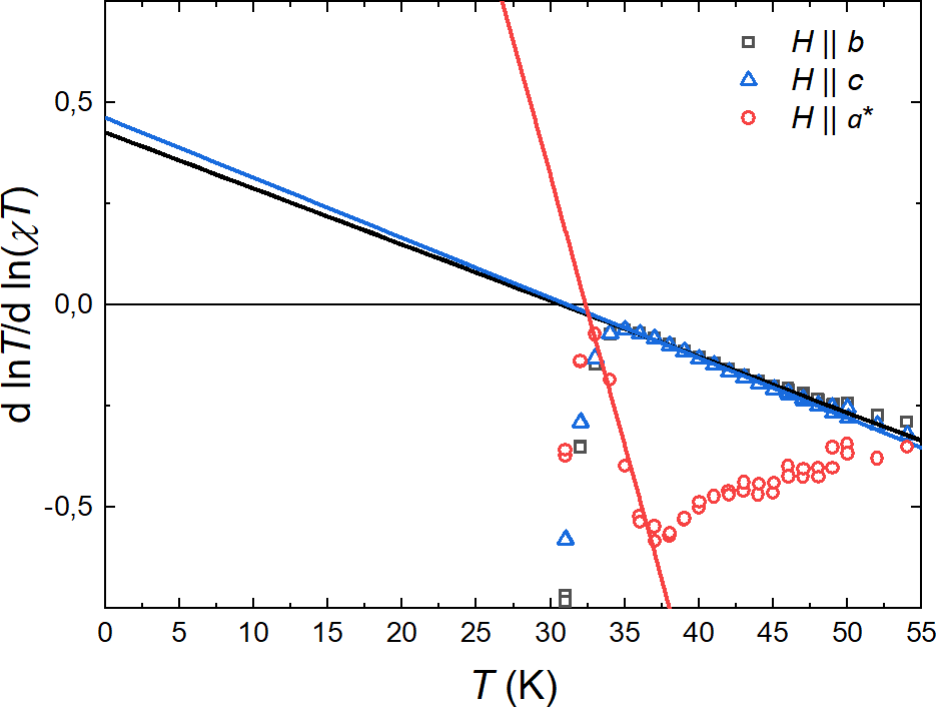
Critical scaling of the magnetic susceptibility. Solid lines show the linear fits to the rescaled χ*T* values.

The ordering process in **1** was analyzed with the static critical scaling of the magnetic susceptibility. To unambiguously obtain the values of γ and *T*_C_, we have used the approach described in a series of papers.^72−75^Figure 13 shows the temperature dependence of the d(ln(*T*))/d(ln(χ*T*)). Linear fits above the phase transition were used to determine γ (the inverse value of the intersection point with the ordinate axis) and *T*_C_ (the intersection with the abscissa axis). The fitting of critical exponents were done between ≈*T*_c_ and ≈1.2*T*_c_ (about 33–40 K) in case of in-plane orientations and between ≈*T*_c_ and ≈1.1*T*_c_ for the hard axis. Both in-plane fits points to high value of the critical exponents γ_*b*_ = 2.34(6) (for *H*||*b*) and γ_*c*_ = 2.16(5) (for *H*||*c*) with similar temperatures of the phase transition *T*_C-*b*_ = 30.8(2) K (for *H*||*b*) and *T*_C-*c*_ = 31.1(2) K (for *H*||*c*). The data for the out-of-plane orientation show a similar value of *T*_C-a*_ = 32.4(2) K; however, the critical exponent for the *H*||a* γ_a*_ = 0.23(2) is 1 order of magnitude lower than for the in-plane orientation. The low value of γ_a*_ points to a very weak temperature dependence of the susceptibility for the hard axis below *T*_C_, which underlines the dominant role of the ordering within the *bc* plane and is symptomatic for 2D magnetism.^71^

**Figure 14:**
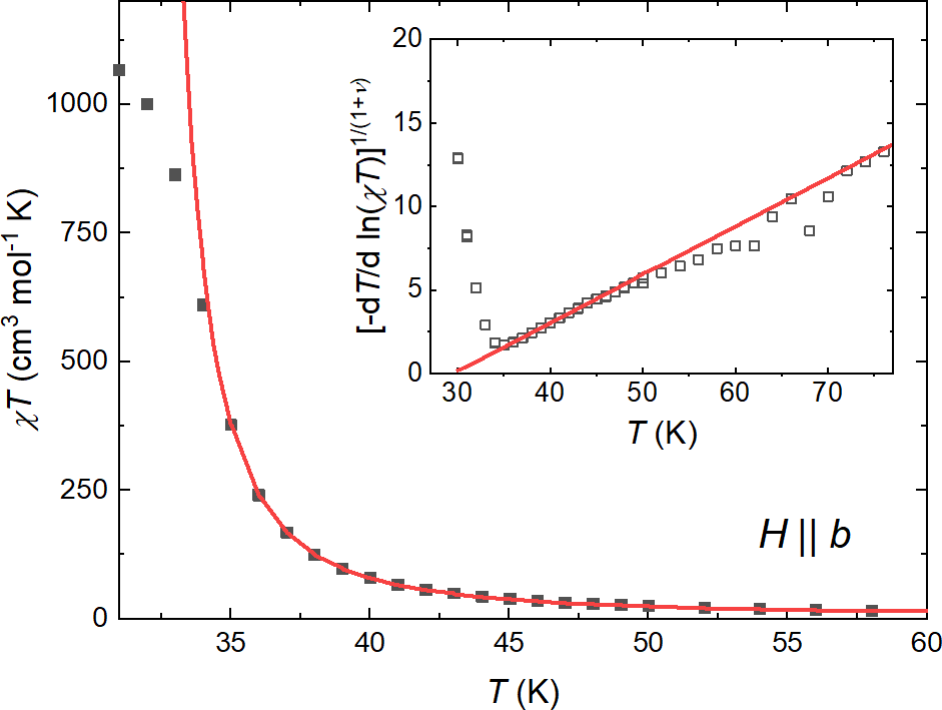
Berezinskii–Kosterlitz–Thouless critical scaling analysis of χ*T* vs *T* for *H*||*b*. Inset shows the indication of the BKT transition for *H*||*b*. The red solid line shows the best fit to χ*T* = *a*_χ_ e^*b*_χ_(*T*–*T*_BKT_)^−ν^^.

The scaling analysis in both in-plane directions (*H*||*b* and *H*||*c*) has revealed high values of γ pointing to 2D ordering process like 2D *XY* (γ = 1.82)^76^ or 2D *XXZ* (γ = 2.17(5))^77^ instead of 3D ones, which are characterized with much lower value of γ (3D Heisenberg γ = 1.385, 3D Ising γ ≈ 1.24 or 3D *XY* γ = 1.32).^78^ The 2D character of the magnetic phase transition can be related to the Berezinskii–Kosterlitz–Thouless topological phase transition in which the bonding of vortex–antivortex pairs occurs below *T*_BKT_ critical temperature.^79−81^ In this case, the magnetic susceptibility follows χ*T* = *a*_χ_ e^*b*_χ_(*T*–*T*_BKT_)^−ν^^ with the critical exponent ν = 0.5. The above equation was used to analyze the susceptibility in both in-plane directions (Figure 14 and S19; the fitting ranges were the same as in the case of γ scaling) revealing: ν_*b*_ = 0.48(5), *T*_BKT-*b*_ = 29.6(3) K, *a*_χ-*b*_ = 1.1(4) emu mol^–1^ K, *b*_χ-*b*_ = 13.1(7) K^ν^ for *H*||*b* and ν_*c*_ = 0.49(3), *T*_BKT-*c*_ = 29.7(1) K, *a*_χ-*c*_ = 1.4(2) emu mol^–1^ K, *b*_χ-*c*_ = 12.6(3) K^ν^ for *H*||*c*. Both obtained values of ν are consistent, within the uncertainty, with the Kosterlitz and Thouless results.

Ferromagnetic 2D ordering within the double-layer and the BKT-type phase transition were also recognized in our previous study of the {(tetrenH_5_)_0.8_Cu^II^_4_[W^V^(CN)_8_]_4_·H_2_O}_*n*_ (**2**) compound which is built of similar and topologically identical Cu^II^–W^V^ bilayers.^71^**2** reveals an LRMO below *T*_C_ ≈ 33 K and the BKT phase transition with *T*_BKT_ = 30.3 K and ν = 0.56(11). The similarity between both compounds is striking: the intrabilayer separation in **1** is ca. 14.0 Å, while in **2** it is only ca. 9.0 Å. Both cases indicate that the significant anisotropy and interactions within the bilayer play the crucial roles and are responsible for the 2D ordering. However, the dipolar interactions have a minor influence on LRMO because even the sizable difference in the separation between the bilayers does not change the basic ordering parameters of both compounds.

## Conclusions

The adeninium AdeH^+^ monocations were successfully used in tuning of interlayer separation between the magnetic coordination {Cu^II^[W^V^(CN)_8_]^−^}_∞_ bilayers in **1**. The cationic layers are composed of 1D infinite hydrogen-bonded ribbons engaging the Hoogsteen face and the sugar face of AdeH^+^ together with the crystallization of H_2_O molecules; one of the topological hydrogen bond synthons existing in the structural database was reproduced in this way. These ribbons are further connected into 2D architecture by orthogonal π–π stacking, to be finally glued to the bimetallic coordination backbone via hydrogen bonds with the terminal CN^–^ bridges. The implementation of such a blocklike arrangement led to the significant increase of separation between {Cu^II^[W^V^(CN)_8_]^−^}_∞_ bilayers, from ca. 9 to ca. 14 Å, compared to the congeners reported previously. Such combinations of polycationic blocks with polynuclear coordination complexes are relatively rare: Some examples are from the chemistry of polyoxometalates,^82,83^ whereas for the first time it is observed in magnetochemistry of polycyanidometalate-based networks.

The complete angle-resolved magnetic measurements confirmed 2D character of magnetic ordering due to a strong magnetic exchange coupling between Cu(II) and W(V) spins within layers, in line with general features observed in the family of {Cu^II^[W^V^(CN)_8_]^−^}_∞_ based compounds. However, several new features were disclosed by this new compound. First, non-negligible magnetic anisotropy was detected within the easy plane, which was not shown experimentally until now. Second, we acquired the nonlinear magnetic susceptibility in a relatively small magnetic field, which was manifested by rare 4-fold symmetry of magnetization detected during the rotation within the easy plane. The observation was rationalized by the calculations using the dedicated model.

The nonlinear magnetic response in a molecular magnet was also investigated by Mito et al. In their research, the [Cr(CN)_6_][Mn(R)-pnH(H_2_O)](H_2_O) compound was measured with means of alternating current susceptibility and higher harmonics. Authors underlined the role of magnetic softness and chirality for the nonlinear response.^84^ In our case, **1** does not reveal chirality; however, the soft magnetic degrees of freedom in the *bc* plane may play the key role in the observed magnetic nonlinear behavior.

Finally, the magnetic {Cu^II^[W^V^(CN)_8_]^−^}_∞_ backbone was incorporated in the crystal of the *C*2 space group, although the total chirality was canceled due to twinning dominated by the component reproduced by inversion symmetry. Nevertheless, further advances toward the general organization of coordination layers in the solid state can be achieved using organic components with diverse distribution of noncovalent interaction generators. The possible coexistence of BKT topological phase transition (within the regime of controlled *XY* magnetism) with chirality (if successfully acquired within the whole crystal) and nonlinear magnetic susceptibility offers the perspectives for systematic research on the magneto-chiral effects on the “colored” coordination backbones of the related type.^85,86^

## Supporting Information Available

Synthesis and basic characterization (materials and syntheses, physical techniques, AdeH^+^ as a supramolecular tecton, SC XRD studies, PXRD studies); magnetic studies (description of all measurement; estimation of the diamagnetic corrections; trigonometric polynomial expansion for the angle-resolved data; scaling analysis); references (PDF)

## Accession Codes

CCDC 2058785 and 2058786 contain the supplementary crystallographic data for this paper. These data can be obtained free of charge via www.ccdc.cam.ac.uk/data_request/cif, or by emailing data_request@ccdc.cam.ac.uk, or by contacting The Cambridge Crystallographic Data Centre, 12 Union Road, Cambridge CB2 1EZ, UK; fax: +44 1223 336033.
