# Supplementary material for: Engineering of the XY Magnetic Layered System with Adeninium Cations: Monocrystalline Angle-Resolved Studies of Nonlinear Magnetic Susceptibility
Source: Inorg Chem. 2021 Jul 7;60(14):10186–98. doi: 10.1021/acs.inorgchem.1c00432 (PMC8388120; doi:10.1021/acs.inorgchem.1c00432)
Supplement: Supplementary file 1 — ic1c00432_si_001.pdf [file ic1c00432_si_001.pdf]

Supporting information

Engineering of the XY Magnetic Layered System with Adeninium Cations: Monocrystalline Angle Resolved Studies of Nonlinear Magnetic Susceptibility

Emilia Kuzniak-Glanowska,[a] Piotr Konieczny*,[b] Robert Pełka,[b] Tadeusz M. Muzioł,[c] Marcin Kozieł,[a] Robert Podgajny*,[a]

[a]*Faculty of Chemistry, Jagiellonian University, Gronostajowa 2, 30-387 Kraków, Poland. E-mail: robert.podgajny@uj.edu.pl*

[b]*Institute of Nuclear Physics PAN, Radzikowskiego 152, 31-342 Kraków, Poland. E-mail: piotr.konieczny@ifj.edu.pl*

[c] *Faculty of Chemistry, Nicolaus Copernicus University in Toruń, Gagarina 7, 87-100 Toruń, Poland.*

Table of content

Synthesis and basic characterization 2
Materials and syntheses 2
Physical techniques 3
AdeH$^+$ as a supramolecular tecton 5
SC XRD studies 10
PXRD studies 10
Magnetic studies 28
Estimation of the diamagnetic contributions 29
Trigonometric polynomial expansion for the angle-resolved data 31
Scaling analysis 33
References 34

Synthesis and basic characterization
Materials and syntheses

$CuCl_2 \cdot 2H_2O$ and adenine were purchased from a commercial source (Aldrich) and used as obtained. $Na_3[W^V(CN)_8]\cdot 4H_2O$ was obtained through the oxidation of $K_4[W^{IV}(CN)_8]$ in the acidic solution (2 M HNO_3) with $KMnO_4$, followed by precipitation of $Ag_3[W(CN)_8]$ with $AgNO_3$ and subsequent metathesis with NaCl in aqueous solution. $K_4[W(CN)_8]$ was prepared according to the literature method.[1] Other chemicals, $NaBH_4$, 30% H_2O_2, $AgNO_3$, KCN concentrated nitric acid and glacial acetic acid were obtained from traditional providers.

$(AdeH)\{Cu^{II}\{W^V(CN)_8\}\}\cdot H_2O$ (**1**) was affordable as slightly elongated hexagonal plate-like single crystals in the result of self-assembly of $Na_3[W(CN)_8)]$, $CuCl_2$ and adenine in acidic aqueous solutions in the dark. Firstly, the freshly prepared solutions of $Na_3[W(CN)_8]\cdot 4H_2O$ (50.0 mg, 0.056 mmol, 4 cm^3 H_2O/HCl – pH = 1) and adenine (14.5 mg, 0.056 mmol, 5 cm^3 H_2O:HCl – pH = 1) were vigorously stirred for 5 minutes. Then, the solution of $CuCl_2 \cdot 2H_2O$ (17.4 mg, 0.056 mmol, 2 cm^3 H_2O/HCl – pH = 1) was slowly added. The mixture was stirred for another 15 minutes and left in the dark to evaporate slowly. After a few weeks, green plates were obtained, separated from the mother liquor and prepared for further analyses. Yield: ~60% Elemental analysis. Calcd (%) for $C_{13}H_{10}CuN_{13}O_2W$ (**1**): C, 24.87; H, 1.61; N, 29.00. Found: C, 24.74; H, 1.631 ; N, 28.90. IR: (cm^{-1}): 3700-2400 *v*(O-H), *v*(N-H) and *v*(C-H) of hydrogen bonded $AdeH^+$; 2206vs, 2192w(sh), 2164 vw(sh), 2153m *v*(C≡N); 1681vs, 1602vs, 1491w, 1463m, 1412s, 1409(sh), 1350m, 1314m, 1236m, 1191m, 1152vw, 1120w, 1002w, 940s, 786mw, 719s, 679m, skeletal vibrations of hydrogen bonded $AdeH^+$; 1637w, 890s vibrations of H_2O molecules (Figure S1). Note that in the range 1650-1600 cm^{-1} and in the range 1000-800 the vibrations of water molecules are expected. The high energy limit of the range of absorption related to *v*(O-H) and *v*(N-H) vibrations is shifted from *ca.* 3430 to *ca.* 3700 cm^{-1} compared to the spectrum of Ade, which is due to the strengthening of hydrogen bond networks. The pattern of the *v*(C≡N) vibrations is in line with those observed previously for the $\{CuW\}^-$ bilayered compounds. [2-5] Some peaks in the fingerprints are notably shifted, which could be explained in terms of protonation of $AdeH^+$ and the specific hydrogen bond pattern. TGA: weight-loss 6.2%; calculated for two molecules of H_2O, 5.7% (Figure S2). The formula was confirmed by the solution and refinement using the synchrotron SCXRD data. PXRD measurements confirmed unequivocally the identity and uniformity of the product among the whole batch of the obtained crystals (Figure S3).

The reference rectangle plate crystals of $(tetrenH_5)_{0.8}\{Cu^{II}[W^{V}(CN)_8]_4\}\cdot 7.2H_2O\}_n$ (**2**) were obtained, identified and indexed according to the protocol reported by us in the literature.[6]

Physical techniques

Elemental analyses of CHN were performed on an Elemental Vario Micro Cube CHNS analyser. Infrared spectra (IR) were measured with crystals or powder samples in the 3500–675 cm^{-1} range using a Nicolet iN10 MX FTIR microscope, in transmission mode. TGA curve TGA studies were carried out by using a Mettler Toledo TGA1 instrument.

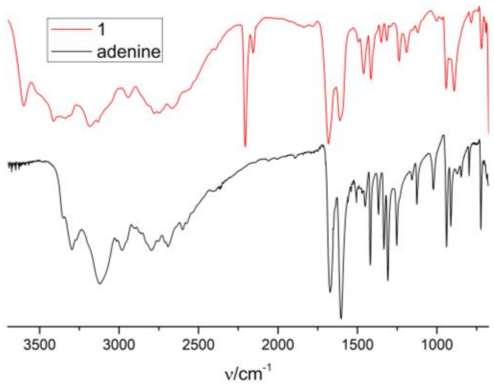

Figure S1. Infrared spectra of adenine (black) and **1** (red).

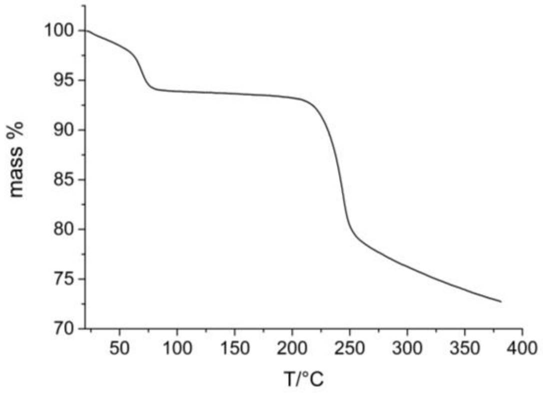

Figure S2. Thermogravimetric analysis for **1**.

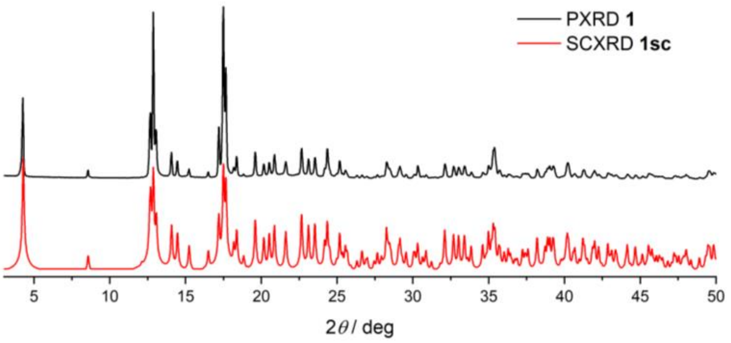

Figure S3. Powder X-ray diffraction patterns in the 3-50° range of 2Θ angle: experimental for **1p** (black line, RT) and calculated from single crystal model of **1** (red line, 100 K).

The monoprotonated form $AdeH^+$ in **1** was postulated considering charge balance and composition involving one singly charged cation per one $\{Cu^{II}[W^{V}(CN)_8]\}^-$ layer unit and two crystallization H_2O, deduced from the magnetic properties and TGA data, respectively. Judging on the form of Ade (Ade, $AdeH^+$ or $AdeH_2^{2+}$) directly form the C-C and C-N bondlengths is rather precluded due to the imperfection of the crystals structure solution and refinements at the region of cationic layer (see also the description of SC XRD studies and the text below the Figure S9). However, the occurrence of the solvent assisted Hoogsteen···Sugar synthon in the literature structures involving exclusively $AdeH^+$ forms support the presence of this form in compound **1**.

$AdeH^+$ as a supramolecular tecton

Adenine and their mono- and doubly protonated cations provide diverse parallel planar or stacked supramolecular synthons. The systematic search of the CSD structural database (completed finally with the ConQuest 2.0.5 on 2020.1 version) involving the specific constrains provided 27 structures with neutral form of adenine (Ade), 67 structures with monoprotonated adeninium cations ($AdeH^+$), and 25 structures involving diprotonated adenine ($AdeH_2^{2+}$) (Figures S4-S6; Tables S1, and S2).

(a)

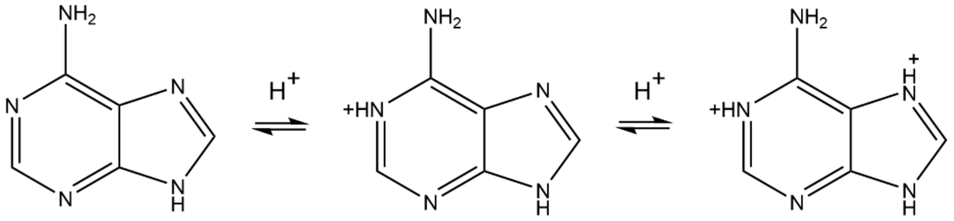

(b)

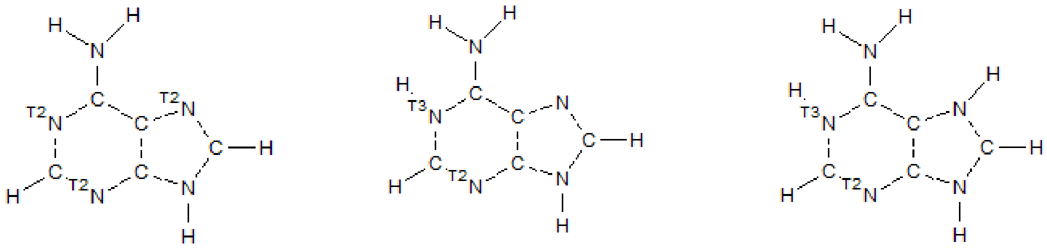

Ade	**$AdeH^+$**	$AdeH_2^{2+}$
27 hits	**67 hits**	25 hits

Figure S4. Protonation equilibria for adenine in aqueous solution (a) and general constrains for the CSD search for the crystal structures Ade, $AdeH^+$ and $AdeH_2^{2+}$ (b). T2 and T3 indicate the number of atoms bonded to the particular atom. Broken lines indicate "any" type of bonds.

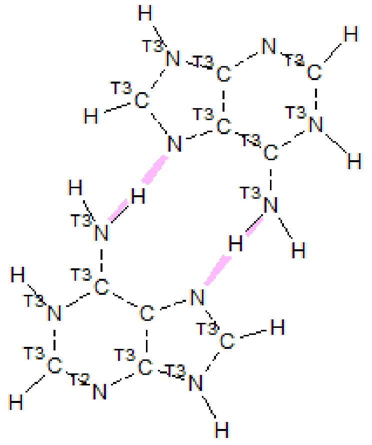

Hoogsteen···Hoogsteen

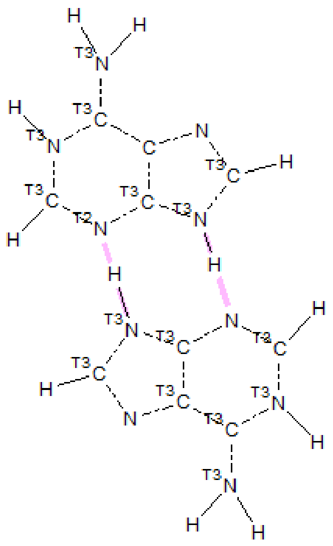

Sugar···Sugar

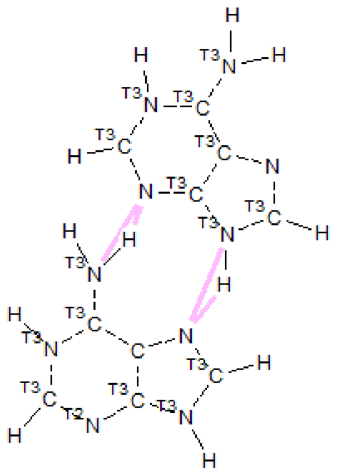

Hoogsteen···Sugar

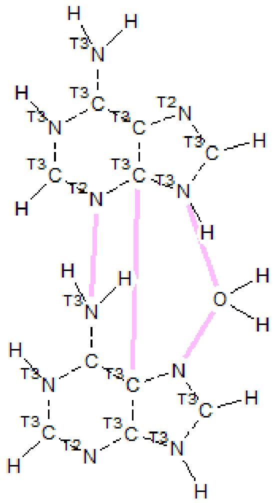

Solvent assisted Hoogsteen···Sugar

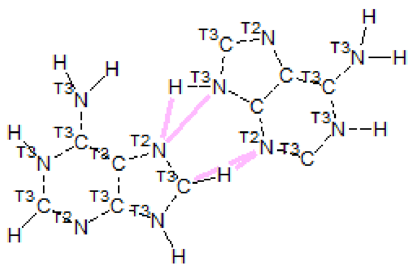

Side Hoogsteen···Sugar

Figure S5. The detailed constrains for the CSD search for various possible in-plane side-to-side synthons between $AdeH^+$ monocations (compare Figure 2b-f). Bold pink lines shows distance contacts that were subjected to the constrains. In general, the suitable N···N separations were limited with the sum of the appropriate VdW radii + 1 Å, whereas the N···H contacts were limited with the sum of the appropriate VdW radii. The obtained sets were further carefully reviewed and intuitively corrected, to achieve reasonable in-plane side-to-side hydrogen bond $AdeH^+\cdots AdeH^+$ contacts. T2 and T3 indicate the number of atoms bonded to the particular atom. Broken lines indicate “any” type of bonds.

Table S1 CCDC refcodes list with information about R factor and presence of disorder in the relative structure – compare with **Figure S5** and **Figure 2**.

CCDC code	**R factor**	**disorder**	**CCCD code**	**R factor**	**disorder**
**Hoogsteen···Hoogsteen**			**Hoogsteen···Hoogsteen, cd.**		
FUBCOX	3.85	yes	VEWFIP	4.48	no
ADENBH	2.5	no	VIGVEM	6.2	yes
ADENCH01	4.6	no	VOFVOE	7.46	no
ADENCH02	6.6	no	**Sugar···Sugar**		
ADENCH03	1.7	no	FUBCOX	3.85	yes
ADENCH04	2.17	no	ADENOH10	2.9	yes
ADENOH10	2.9	yes	ADENPH	6.8	no
ADESUL	6.7	no	ADESUL	6.7	no
APUHEZ	6.83	yes	BUDJAM	3.2	no
AYIMEC	2.88	no	IWOTOF	2.97	yes
BETNOD	8.5	yes	KEVGAW	4.9	no
COTTIQ	3.95	no	LOSHUY	3.03	no
IKAFOQ	3.92	yes	QUTKEW	6.58	yes
IWOTOF	2.97	yes	ROPMIU	3.11	no
KEVGAW	4.9	no	SEQVAN	5.52	no
LEZHIH	5.5	no	SIYBAF	5.54	yes
LOLDAS	5.12	no	UDIJEX	4.95	no
LOSHUY	3.03	no	UWAMUC	4.23	no
MUCCOD	2.67	no	VEWFIP	4.48	no
PANSAA	5.44	no	VIGVEM	6.2	yes
RIGMEA	4.64	no	**Hoogsteen···Sugar**		
RIGMIE	4.38	no	LICGIO	3.99	no
ROPMIU	3.11	no	**Solvent assisted Hoogsteen···Sugar**		
SIXZUW	5.98	yes	EGOWIG	4.21	no
SIYBAF	5.54	yes	LOLDEW	5.41	no
SOLDAZ	2.73	no	LOLDIA	4.64	yes
SOLDAZ01	3.13	no	UWAMEM	5.89	no
TINYIZ	3.49	no	UWAMEM01	6.62	no
TUDLOT	4.5	no	**Side Hoogsteen···Sugar**		
UDIJEX	4.95	no	KEVGEA	5.24	No
UWAMEM01	6.62	no	SEQTUF	3.45	No

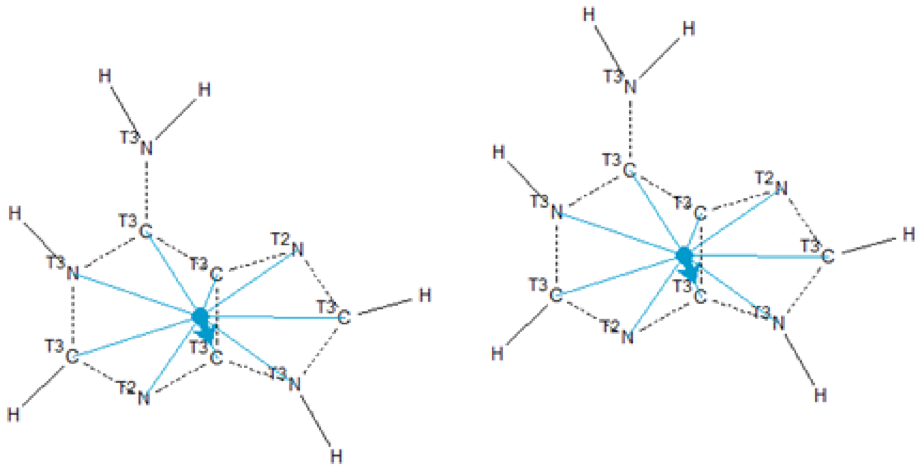

(top)

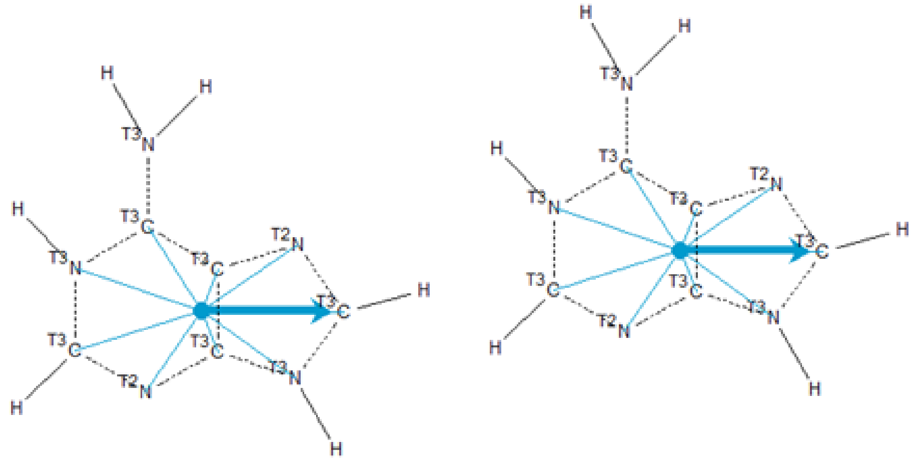

(bottom)

Figure S6. The detailed constrains for the CSD search for various general stacked synthons between AdeH$^+$ monocations (compare **Figure 3a-d**). The angle between planes defined by the rings' atoms were set in range 0-15 deg. Intermolecular distance defined by a vectors between the centroids of the AdeH$^+$ rings (blue points below) in contact were set in range 2.5-4.3 Å. The above filters allowed for selection of parallel (or almost parallel), stack-like synthons AdeH$^+$··· AdeH$^+$ synthons. Further distinction required careful analysis of angles between vectors defined by centroids and atoms C4 and C8 respectively. For example: if angles between both vectors defined as centroid–C4 in two neighboring AdeH$^+$ cations (top section below) and vectors defined by centroid–C8 molecules in two neighboring AdeH$^+$ cations (bottom section below) are in range 165-180 deg, then this pair of molecules was assigned to group (a) in Figure 3 in the main text). Accordingly, both angles between 0 and 15 deg constituted group (b). For the group (c) both angles were carefully considered, either. T2 and T3 indicate the number of atoms bonded to the particular atom. Broken lines indicate "any" type of bonds.

Table S2 CCDC refcodes list with information about R factor and presence of disorder in the relative structure – compare with Figure S6 and Figure 3 (main text)

CCDC code	R-factor	disorder	CCDC code	R-factor	disorder
**Figure 3a**			**Figure 3a cd**		
FUBCOX	3.85	yes	UDIJEX	4.95	no
ADENOH10	2.9	yes	VEWFIP	4.48	no
ADESUL	6.7	no	VIGVEM	6.2	yes
APUHEZ	6.83	yes	VITMES	4.03	no
BETNOD	8.5	yes	**Figure 3b**		
EGOWIG	4.21	no	FUBCOX	3.85	yes
EVIFIY	4.21	no	BOTSIN	3.74	yes
LEZHIH	5.5	no	SEQVAN	5.52	no
GADPOS	6.97	no	SIXZUW	5.98	yes
LOLDIA	4.64	yes	SIYBAF	5.54	yes
LOSHUY	3.03	no	XIVBOV	8.77	no
PANSAA	5.44	no	**Figure 3c**		
ROPMIU	3.11	no	KEVGAW	4.9	no

SC XRD studies

Data collection for (**1**) was performed on BESSY II synchrotron BL14-3 beamline (Helmholtz Zentrum Berlin, Bessy II) with radiation wavelength λ = 0.89429 Å at 100 K. The data were processed using both *xdsapp*[7,8] and CrysAlis Pro[9] to take into account an absorption correction. The structure was solved by direct methods using SHELXS program, and the structural models were refined by full-matrix least squares technique (SHELX 2018/1).[10] All non-hydrogen atoms were refined anisotropically. Positions of hydrogen atoms were assigned at calculated positions with displacement parameters being 20% higher than corresponding carbon atoms. Hydrogen atom at N13 and N17 were located due to difference electron density maps and assigned with thermal displacement parameters 20% higher than corresponding nitrogen atoms. Analysis performed in WinGX[11] showed twinning (ROTAX program) and allowed for assignment of proper twinning matrix which was subsequently used in SHELX program with proper BASF and HKLF 4 flags. To assure stable refinement and reasonable model several geometrical restraints were applied mainly for $(adeH)^+$ cation (DFIX, DANG, FLAT), whereas restraints for thermal displacement parameters (ISOR) were applied for cyanide anions. In the final model due to quality of the electron density map there are missing hydrogen atoms attached to crystallization O31 and O32 water molecules and N11 NH_2 group of $AdeH^+$.

PXRD studies

Powder diffraction pattern of **1** for structure determination was collected using Panalytical X'pert PRO MPD diffractometer in Debye-Scherrer geometry. The data were collected using copper X-ray source ($\lambda_{CuK\alpha}$ = 1.5419 Å) over the range of 3 – 85° 2θ at room temperature. The pattern was indexed using N-Treor routine in EXPO2014 program.[12] The structure was solved using the EXPO2014 program yielding positions of metal centres reproducing the arrangement of double layers known for the parent structure.[2] Due to the large unit cell volume and the presence of heavy elements, the rest of the structural information remained garbled. The next step of structure determination was done by means of FOX program.[13] The bridging cyanido ligands were introduced in geometrical positions between metal centres accordingly to typical W-C-N-Cu geometry. Three terminal CN groups were inserted at the apical position of tungsten atom and restrained, leaving one rotational degree of freedom. Detailed analysis of Cambridge Structural Database (CSD)[14] regarding all entries containing $AdeH^+$ ion revealed that in the absence of strong hydrogen bond donors or acceptors (like carboxylic groups), the prevalent structural motif is the centrosymmetric $R_2^2(10)$ synthon. Consequently, a dimeric adenine

model was prepared (without hydrogen atoms) and introduced into the model structure in one of the four accessible inversion centres, with constrained geometry and rotational degrees of freedom. For each adenine position 0, 1 or 2 water molecules (of expected two molecules, basing on composition) were included in the model. Each of the starting models was optimized and the best results were subsequently refined using Jana2006 software.[15]

Table S3 Crystal data and structure refinement of **1** and **1p** solutions.

Compound	1	1p
**Formula**	$C_{13}H_{10}CuN_{13}O_2W$	$C_{26}H_{20}Cu_2N_{26}O_4W_2$
**Formula weight [g·mol^{-1}]**	627.73	1255,38
**Temperature [K]**	100.0	293
**Crystal system**	monoclinic	Triclinic
**Space group**	C2	$P\bar{1}$
**Unit cell**		
**a [Å]**	41.3174(12)	7.316(2)
**b [Å]**	7.0727(3)	20.938(7)
**c [Å]**	7.3180(2)	7.072(2)
**α, β, γ [deg]**	90, 93.119(3), 90	99.67(5), 89.97(5), 86.92(3)
**Volume [Å^3]**	2135.34	1066.2(6)
**Z**	4	1
**ρ_{calc} [g/cm^3]**	1.953	
**Absorption coefficient [mm^{-1}]**	11.308	
**F(000)**	1192.0	
**Crystal size [mm^3]**	0.170 × 0.140 × 0.070	-
**Radiation [Å]**	synchrotron (λ = 0.89429)	
**2Θ range [deg]**	7.016 to 67.946	
***hkl* ranges**	$-51 \le h \le 51$, $-7 \le k \le 7$, $-9 \le l \le 9$	
**Reflections collected**	13946	
**Independent reflections**	4022 [R_{sigma} = 0.0255]	
**R_{int}**	0.0322	
**Data/restraints/ parameters**	4022/98/274	
**GOOF on F^2**	1.119	
**Final R indexes [I>=2σ (I)]**	R1 = 0.0341, wR2 = 0.0908	
**Final R indexes [all data]**	R1 = 0.0358, wR2 = 0.0925	
**Largest diff. peak/hole [e · Å^{-3}]**	2.53/-1.50	

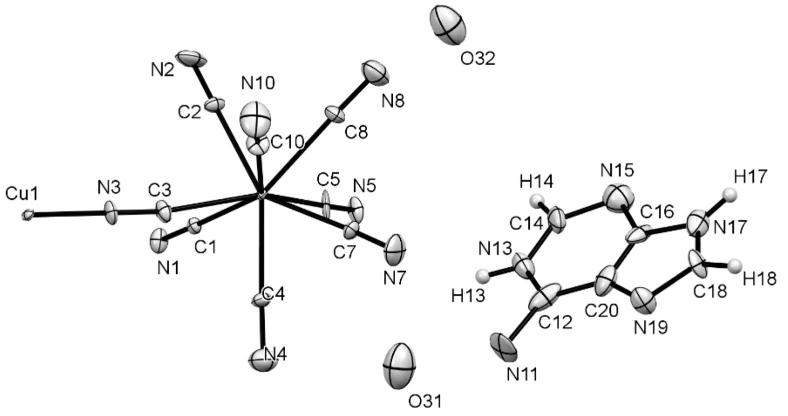

Figure S7. Asymmetric unit in the **1** model. Thermal ellipsoid for **1** are drawn at the 30% probability level.

The bond lengths and angles along the C,N backbone of the $AdeH^+$ cations in structural model **1** are in a reasonable agreement with the data shown previously in literature (Figures S8, and S9 and Table S7). Some authors reported that protonation state of adeninium cations can be specified by careful analysis of geometrical parameters e.g. bond length and angles. [16-18] Unfortunately, some imperfections in adenine atoms position determination occurring as the result of weak diffraction vanishing during the experiment time and imperfect absorption correction related to plenty of heavy atoms in structure, preclude the reliable correlation in **1**. Nevertheless, we postulate the monoprotonated state of adenine, as the charge balance in the considered structure model is maintained only with the one monocharged $AdeH^+$ cation per one $\{Cu^{II}[W^{V}(CN)_8]\}^-$ layer unit and two crystallization H_2O, the former condition assured by the observed magnetic properties and the second condition derived from TGA data. The assumed protonation of N^1 atom ($^+$N1-H) and the presence of the additional proton attached to the N^9 (N-H) is suggested according to general notion based on the numerous experimental and theoretical works,[16-21] and consistent with the proximity of the O31 atom and O32 atom, respectively, as potential proton acceptors.

Table S4. The bond length [Å] and angles [deg] in the model **1.**

			**Bond length [Å]**				
W1	C1	Single	2.172(8)	N7	C7	Triple	1.102(18)
W1	C2	Single	2.130(17)	N8	C8	Triple	1.15(2)
W1	C3	Single	2.170(18)	N10	C10	Triple	1.151(16)
W1	C4	Single	2.09(2)	N11	C12	Single	1.44(2)
W1	C5	Single	2.20(2)	C12	N13	Aromatic	1.301(19)
W1	C8	Single	2.157(14)	C12	C20	Aromatic	1.33(3)
W1	C10	Single	2.148(11)	N13	H13	Single	0.8800
W1	C7	Single	2.169(11)	N13	C14	Aromatic	1.308(17)
Cu1	N3	Single	1.919(17)	C14	H14	Single	0.9500
Cu1	N1	Single	2.129(8)	C14	N15	Aromatic	1.358(13)
Cu1	N2	Single	2.04(2)	N15	C16	Aromatic	1.40(2)
Cu1	N4	Single	1.97(2)	C16	N17	Single	1.33(2)
Cu1	N5	Single	1.980(16)	C16	C20	Aromatic	1.32(2)
N1	C1	Triple	1.139(12)	N17	H17	Single	0.8800
N2	C2	Triple	1.11(3)	N17	C18	Single	1.31(2)
N3	C3	Triple	1.16(2)	C18	H18	Single	0.9500
N4	C4	Triple	1.19 (3)	C18	N19	Double	1.31(2)
N5	C5	Triple	1.09(3)	N19	C20	Single	1.39(2)
			**Angle [°]**				
C1	W1	C10	139.6(5)	C5	W1	C1	74.6(8)
C1	W1	C8	124.9(10)	C5	W1	C10	141.4(7)
C1	N1	Cu1	177(2)	C5	W1	C8	68.1(7)
C10	W1	C8	75.1(5)	C5	N5	Cu1	173.2(19)
C12	N13	C14	126(2)	C7	W1	C2	141.8(6)
C12	N13	H13	117.0	C7	W1	C5	82.6(6)
C12	C20	N19	135(2)	C7	W1	C1	139.3(5)
C12	C20	C16	115.7(18)	C7	W1	C10	76.3(5)
C14	N15	C16	110.0(16)	C7	W1	C8	73.9(5)
C14	N13	H13	117.0	N1	C1	W1	178(2)
C16	N17	H17	127.2	N10	C10	W1	176.5(16)
C18	N19	C20	101.8(16)	N13	C14	N15	121.2(19)
C18	N17	C16	105.7(18)	N13	C14	H14	119.4
C18	N17	H17	127.2	N13	C12	N11	120.3(19)
C2	W1	C5	94.5(8)	N15	C14	H14	119.4
C2	W1	C1	74.3(8)	N17	C18	N19	114.8(13)
C2	W1	C10	83.1(6)	N17	C18	H18	122.6
C2	W1	C8	69.9(6)	N17	C16	C20	108.0(17)
C2	N2	Cu1	167(2)	N17	C16	N15	123(2)
C20	C16	N15	128.8(17)	N19	C20	C16	109.7(16)
C20	C12	N13	118.1(19)	N19	C18	H18	122.6
C20	C12	N11	121.6(17)	N2	Cu1	N4	160.6(9)
C3	W1	C7	119.3(6)	N2	Cu1	N1	99.5(9)

C3	W1	C2	82.5(6)	N2	C2	W1	175(2)
C3	W1	C5	147.1(7)	N3	Cu1	N2	89.6(7)
C3	W1	C1	73.1(8)	N3	Cu1	N4	94.9(10)
C3	W1	C10	71.0(6)	N3	Cu1	N1	100.2(8)
C3	W1	C8	138.3(7)	N3	C3	W1	172.3(19)
C3	N3	Cu1	175.3(17)	N4	Cu1	N1	98.3(9)
C4	W1	C3	82.3(8)	N4	C4	W1	175(2)
C4	W1	C7	72.6(7)	N5	Cu1	N3	158.2(7)
C4	W1	C2	144.9(8)	N5	Cu1	N2	82.0(10)
C4	W1	C5	81.7(5)	N5	Cu1	N4	87.0(6)
C4	W1	C1	71.0(9)	N5	Cu1	N1	101.1(8)
C4	W1	C10	120.9(7)	N5	C5	W1	176.5(19)
C4	W1	C8	137.1(7)	N7	C7	W1	176.4(14)
C4	N4	Cu1	174(2)	N8	C8	W1	177.7(15)

Table S5. Results of Continuous Shape Measures Analysis for tungstate and copper coordination centers basing on **1** model). The existing coordination polyhedral are marked in bold, and are typical for all compounds built of $\{Cu^{II}[W^{V}(CN)_8\}^-$ backbones.

Centre	Geometry		Geometry	
**W**	OP-8	29.746	JGBF-8	14.138
	HPY-8	24.083	JETBPY-8	27.913
	HBPY-8	16.401	JBTPR-8	1.627
	CU-8	11.309	**BTPR-8**	**0.763**
	SAPR-8	1.210	JSD-8	4.095
	TDD-8	1.801	TT-8	11.994
**Cu**	PP-5	30.869	**SPY-5**	**0.288**
	vOC-5	1.343	JTBPY-5	7.531
	TBPY-5	5.055		

OP-8 - Octagon
HPY-8 - Heptagonal pyramid
HBPY-8 - Hexagonal bipyramid
CU-8 - Cube
SAPR-8 - Square antiprism
TDD-8 - Triangular dodecahedron
JGBF-8 - Johnson gyrobifastigium J26
JETBPY-8 - Johnson elongated triangular bipyramid J14
JBTPR-8 - Biaugmented trigonal prism J50
BTPR-8 - Biaugmented trigonal prism/Bicapped trigonal prism
JSD-8 - Snub diphenoid J84
TT-8 - Triakis tetrahedron
PP-5 – Pentagon
vOC-5 - Vacant octahedrons
TBPY-5 - Trigonal bipyramid
SPY-5 - Spherical square pyramid
JTBPY-5 - Johnson trigonal bipyramid J12

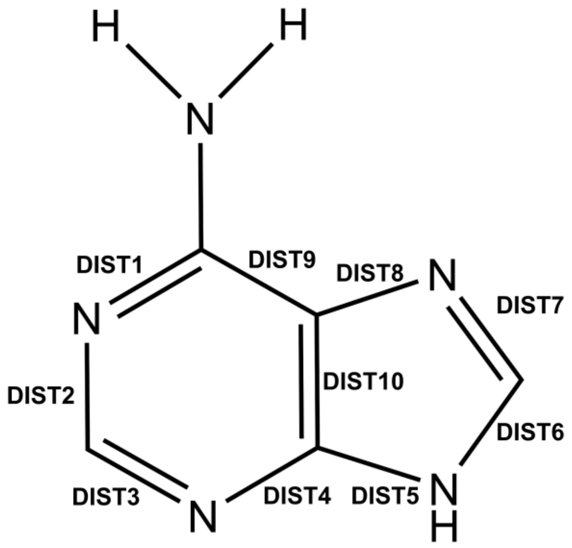

Figure S8. Bond labels in adenine skeleton. For comparison of the data see histograms in Figure S9 and Table S6.

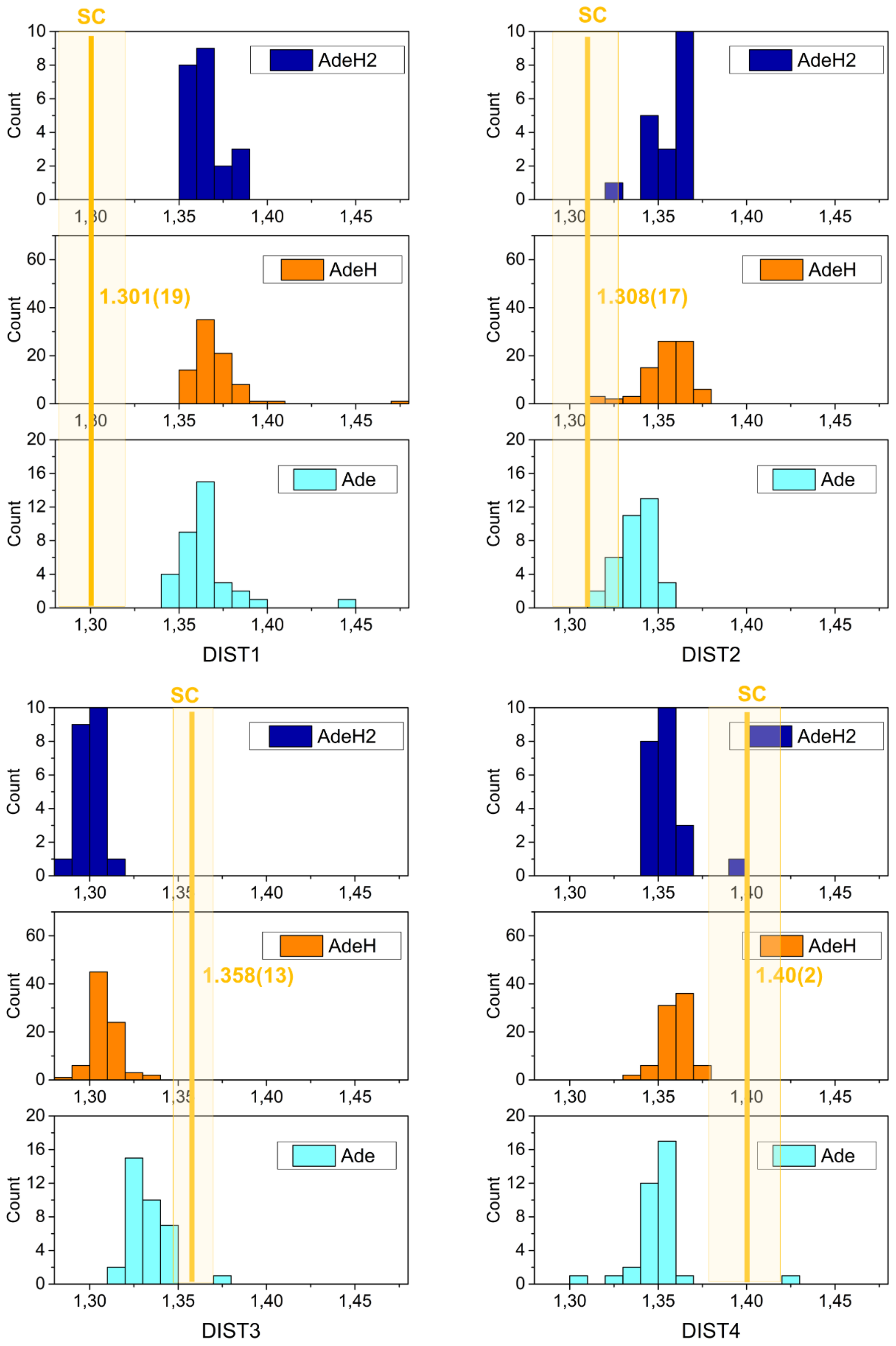

Figure S9 The comparison of the C-C and C-N bond lengths of $AdeH^+$ cation in the structural model **1** (yellow lines and pale yellow shaded areas illustrating the standard deviations of the distances in **1**) with those found for the neutral Ade, monoprotonated $AdeH^+$ and doubly protonated $AdeH^{2+}$ in the CSD database. See the Figure S8 for the bond lengths numbering.

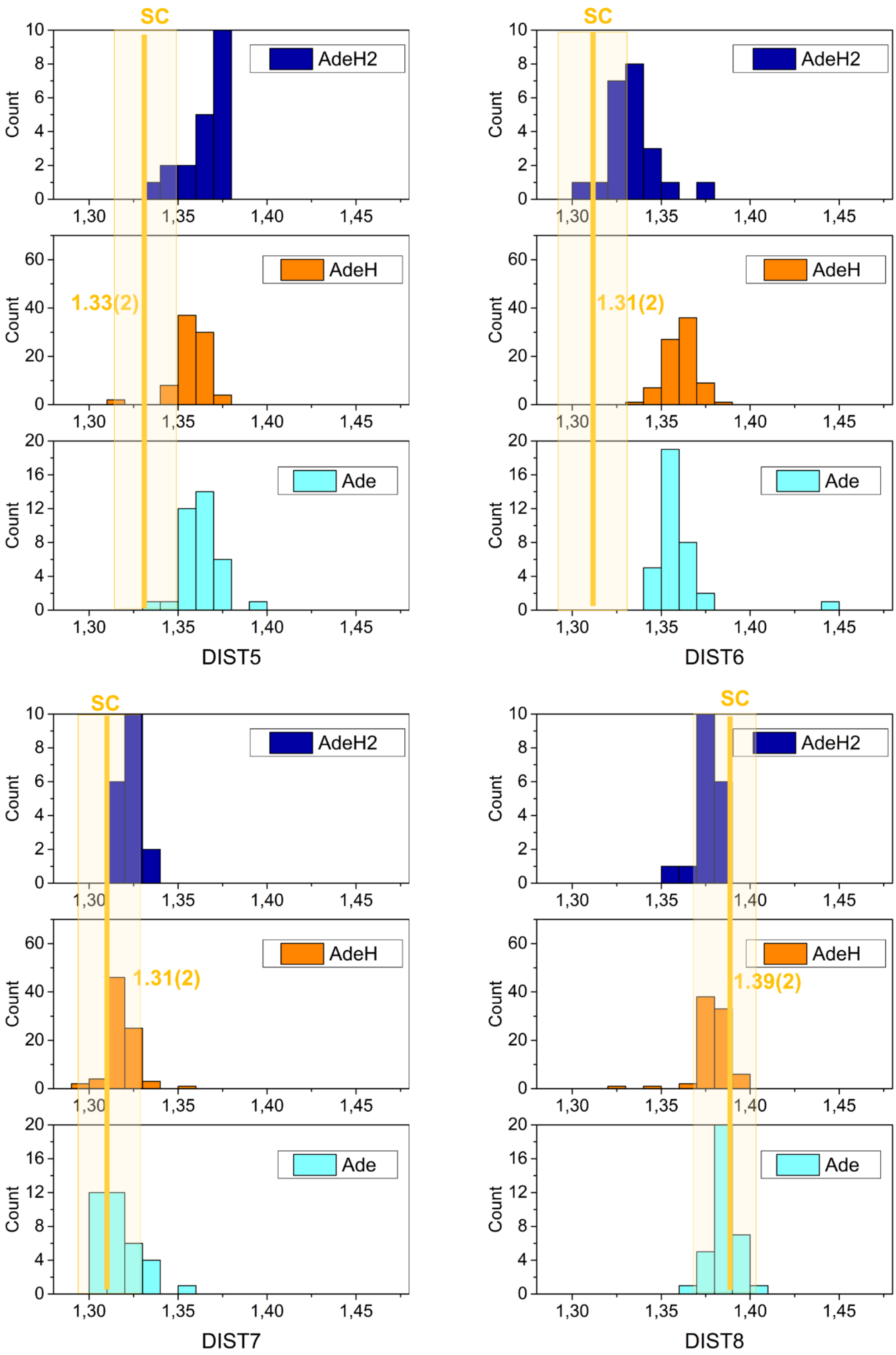

Figure S9 continued.

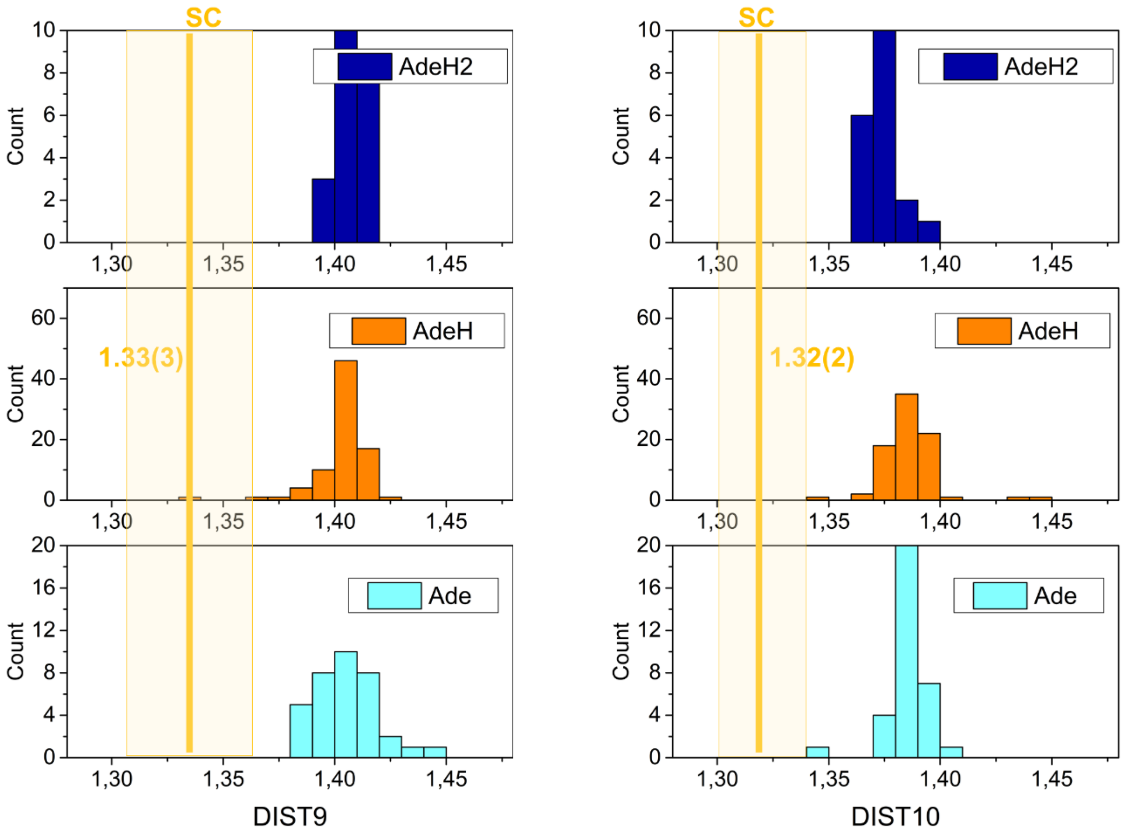

Figure S9 continued.

The yellow vertical lines in Figure S9 indicate the C-C and C-N bond lengths in organic molecule found in SC XRD model **1**, whereas the yellow-shaded bars presents the standard deviations. The information provided by each separate part DISTX may indicate the occurrence of one particular protonation form, however their combination does not create the consistent image. The same conclusion appears when one consider only the appropriate six distances sets found the 5 structures (EGOWIG, LOLDEW, LOLDIA, UWAWEM, UWAWEM1) showing the similar H-bonds patterns (Figure 2e, main text). We found, however, that the considered flat motif was found practically only in the structures containing monoprotonated $AdeH^+$ species. One additional motif based on doubly protonated $AdeH_2^{2+}$ form was found in the structure DUJHUN, however, in this case the $AdeH_2^{2+}$ dications do not share the same plane. The distribution of C-C and C-N distances in DUJHUN is very similar to those found in abovementioned $AdeH^+$ monocations. We hope then that the observed preference can support the exclusive presence of $AdeH^+$ forms in the crystal structure of **1**, despite some imperfection of non-corrected positions of non-hydrogen atoms in our $AdeH^+$ forms.

Table S6 All records from CCDC database used to prepare bond lengths histograms in Figure S8 together with R-factors and information about disorder in structure.

**CCDC code**	**DIST1**	**DIST10**	**DIST2**	**DIST3**	**DIST4**	**DIST5**	**DIST6**	**DIST7**	**DIST8**	**DIST9**	**R-factor**	**disorder**
						**Ade**						
NOZQUR	1.365	1.383	1.332	1.335	1.349	1.359	1.36	1.305	1.38	1.41	5.52	yes
BERNAP	1.373	1.388	1.327	1.337	1.352	1.355	1.355	1.318	1.388	1.391	6.45	no
BIGHIJ	1.362	1.383	1.336	1.333	1.346	1.371	1.354	1.305	1.393	1.405	4.54	no
COFBUU10	1.345	1.396	1.345	1.338	1.342	1.358	1.375	1.312	1.381	1.401	5.7	no
EVIFIY	1.355	1.387	1.348	1.329	1.355	1.357	1.359	1.316	1.387	1.408	4.21	no
FESWUW	1.353	1.383	1.38	1.284	1.357	1.357	1.37	1.297	1.389	1.389	3.91	yes
FUSVAQ01	1.36	1.393	1.343	1.331	1.348	1.368	1.359	1.324	1.393	1.414	5.0	no
GUNQUB	1.367	1.395	1.349	1.342	1.351	1.379	1.371	1.325	1.393	1.419	2.55	no
IWOTOF	1.367	1.388	1.35	1.319	1.354	1.367	1.359	1.315	1.38	1.405	2.97	yes
JOZZED	1.347	1.381	1.343	1.324	1.35	1.371	1.357	1.316	1.395	1.411	4.71	no
KOBFUD	1.368	1.381	1.351	1.321	1.35	1.367	1.357	1.304	1.378	1.421	8.53	no
KOBFUD	1.371	1.397	1.344	1.344	1.339	1.357	1.353	1.329	1.375	1.39	8.53	no
KOBFUD01	1.368	1.381	1.35	1.32	1.35	1.367	1.357	1.304	1.379	1.42	6.8	no
KORGUV	1.441	1.409	1.325	1.374	1.367	1.336	1.352	1.359	1.379	1.391	8.88	no
LAFSUH	1.35	1.376	1.341	1.324	1.345	1.363	1.355	1.307	1.389	1.398	4.76	no
LAFSUH	1.352	1.379	1.331	1.323	1.343	1.367	1.349	1.313	1.388	1.406	4.76	no
LAFTAO	1.345	1.385	1.34	1.34	1.338	1.377	1.361	1.316	1.386	1.403	4.25	no
LAFTAO	1.356	1.383	1.324	1.333	1.357	1.361	1.354	1.324	1.4	1.399	4.25	no
LOLCUL	1.363	1.388	1.336	1.337	1.353	1.364	1.361	1.317	1.386	1.41	4.62	no
LOLCUL	1.368	1.387	1.331	1.334	1.35	1.365	1.359	1.315	1.391	1.406	4.62	no
MUBRAD	1.357	1.392	1.341	1.324	1.348	1.364	1.357	1.317	1.385	1.416	4.07	yes
NILNUS	1.366	1.386	1.335	1.322	1.355	1.357	1.365	1.304	1.389	1.395	4.0	no
OYEFUU	1.354	1.389	1.33	1.327	1.342	1.362	1.346	1.309	1.388	1.402	4.97	no
OYEFUU	1.362	1.377	1.346	1.324	1.345	1.367	1.365	1.308	1.387	1.405	4.97	no
QOTFOV	1.35	1.348	1.328	1.345	1.422	1.363	1.449	1.331	1.381	1.448	5.92	yes

SEQVIV	1.362	1.382	1.34	1.325	1.352	1.37	1.351	1.324	1.39	1.407	5.86	no
TAZWOH	1.38	1.389	1.329	1.329	1.341	1.353	1.349	1.304	1.378	1.382	6.99	yes
TAZWOH01	1.362	1.383	1.339	1.327	1.356	1.367	1.354	1.316	1.389	1.41	4.15	no
UWAMUC01	1.367	1.39	1.361	1.307	1.354	1.353	1.352	1.324	1.372	1.403	5.96	no
VILLIM	1.345	1.371	1.338	1.319	1.355	1.357	1.356	1.309	1.388	1.398	6.86	no
VOFVOE	1.378	1.388	1.338	1.34	1.358	1.352	1.364	1.332	1.386	1.389	7.46	no
VOFVOE	1.38	1.389	1.325	1.337	1.356	1.354	1.355	1.328	1.391	1.387	7.46	no
WAWRET	1.356	1.383	1.347	1.328	1.346	1.373	1.365	1.314	1.383	1.417	2.11	yes
WUPGOE	1.36	1.391	1.317	1.324	1.348	1.345	1.345	1.309	1.369	1.43	14.72	no
WUPGOE	1.39	1.384	1.347	1.342	1.306	1.399	1.357	1.336	1.381	1.381	14.72	no
WUPGUK	1.354	1.386	1.333	1.348	1.351	1.354	1.365	1.302	1.388	1.384	9.49	no
WUPGUK	1.362	1.399	1.316	1.335	1.326	1.354	1.342	1.338	1.38	1.392	9.49	no
						**$AdeH^+$**						
FUBCOX	1.364	1.386	1.369	1.313	1.362	1.357	1.366	1.322	1.383	1.415	3.85	yes
FUBCOX	1.367	1.389	1.354	1.313	1.364	1.357	1.37	1.316	1.389	1.405	3.85	yes
FUBCOX	1.368	1.392	1.355	1.313	1.36	1.356	1.369	1.315	1.381	1.413	3.85	yes
ADALEW	1.396	1.396	1.329	1.301	1.367	1.314	1.361	1.298	1.347	1.363	6.21	no
ADALEW	1.473	1.44	1.311	1.318	1.338	1.317	1.351	1.328	1.329	1.331	6.21	no
ADENBH	1.366	1.378	1.371	1.309	1.363	1.357	1.355	1.316	1.382	1.408	2.5	no
ADENCH01	1.384	1.372	1.37	1.301	1.358	1.359	1.33	1.353	1.372	1.401	4.6	no
ADENCH02	1.364	1.377	1.369	1.289	1.358	1.359	1.351	1.32	1.377	1.403	6.6	no
ADENCH03	1.364	1.394	1.371	1.308	1.353	1.361	1.365	1.323	1.38	1.408	1.7	no
ADENCH04	1.363	1.392	1.369	1.306	1.354	1.36	1.365	1.322	1.379	1.408	2.17	no
ADENOH10	1.361	1.382	1.365	1.302	1.361	1.354	1.358	1.313	1.386	1.406	2.9	yes
ADENOH10	1.363	1.39	1.349	1.303	1.353	1.348	1.366	1.31	1.372	1.404	2.9	yes
ADENPH	1.367	1.364	1.35	1.305	1.37	1.357	1.355	1.306	1.379	1.429	6.8	no
ADESUL	1.371	1.378	1.358	1.313	1.364	1.363	1.356	1.325	1.395	1.409	6.7	no
ADESUL	1.389	1.374	1.351	1.309	1.363	1.366	1.362	1.314	1.388	1.414	6.7	no
ADHCOS10	1.381	1.375	1.36	1.307	1.361	1.35	1.368	1.318	1.378	1.396	6.0	no
APUHEZ	1.377	1.386	1.365	1.306	1.371	1.366	1.375	1.327	1.393	1.417	6.83	yes

APUHEZ	1.384	1.395	1.372	1.306	1.375	1.37	1.381	1.318	1.392	1.405	6.83	yes
AYIMEC	1.371	1.393	1.355	1.304	1.354	1.352	1.357	1.317	1.378	1.396	2.88	no
BETNOD	1.358	1.374	1.361	1.308	1.362	1.367	1.366	1.325	1.386	1.414	8.5	no
BETNOD	1.37	1.377	1.366	1.314	1.366	1.355	1.364	1.318	1.39	1.407	8.5	yes
BOTSIN	1.365	1.377	1.359	1.301	1.358	1.361	1.35	1.315	1.383	1.412	3.74	yes
BUDJAM	1.369	1.382	1.361	1.308	1.363	1.362	1.362	1.322	1.38	1.41	3.2	no
BUDVUR10	1.364	1.391	1.347	1.304	1.364	1.377	1.36	1.31	1.379	1.387	5.3	no
COFBOO10	1.356	1.396	1.373	1.293	1.339	1.373	1.356	1.317	1.378	1.408	8.5	yes
COTTIQ	1.386	1.391	1.347	1.314	1.35	1.352	1.364	1.31	1.367	1.403	3.95	no
EGOWIG	1.375	1.39	1.34	1.308	1.351	1.356	1.354	1.312	1.377	1.384	4.21	no
EVIFIY	1.362	1.386	1.356	1.307	1.365	1.351	1.362	1.314	1.384	1.408	4.21	no
GADPOS	1.368	1.388	1.348	1.31	1.368	1.353	1.366	1.311	1.385	1.387	6.97	no
IKAFOQ	1.365	1.385	1.369	1.302	1.357	1.362	1.361	1.321	1.384	1.408	3.92	yes
IWOTOF	1.373	1.382	1.359	1.303	1.368	1.353	1.366	1.323	1.376	1.407	2.97	yes
IWOTOF	1.378	1.386	1.354	1.308	1.36	1.359	1.367	1.319	1.379	1.401	2.97	yes
KEVGAW	1.365	1.392	1.365	1.314	1.364	1.367	1.371	1.323	1.38	1.411	4.9	no
KEVGEA	1.362	1.385	1.359	1.31	1.358	1.364	1.36	1.317	1.379	1.418	5.24	no
LEZHIH	1.358	1.373	1.356	1.316	1.365	1.368	1.353	1.33	1.384	1.408	5.5	no
LEZHIH	1.367	1.386	1.35	1.321	1.349	1.367	1.371	1.317	1.381	1.408	5.5	no
LICGIO	1.359	1.379	1.342	1.311	1.357	1.362	1.353	1.313	1.387	1.409	3.99	no
LICGIO	1.381	1.383	1.347	1.302	1.362	1.35	1.361	1.314	1.376	1.4	3.99	no
LOLDAS	1.366	1.392	1.361	1.311	1.355	1.359	1.361	1.312	1.387	1.4	5.12	no
LOLDEW	1.364	1.394	1.34	1.31	1.36	1.377	1.36	1.328	1.384	1.408	5.41	no
LOLDIA	1.374	1.392	1.36	1.307	1.357	1.36	1.36	1.319	1.376	1.402	4.64	yes
LOLDOG	1.366	1.388	1.353	1.307	1.359	1.361	1.366	1.319	1.379	1.415	5.72	no
LOSHUY	1.368	1.38	1.354	1.302	1.363	1.36	1.364	1.315	1.389	1.408	3.03	no
MUCCOD	1.363	1.392	1.365	1.308	1.357	1.362	1.363	1.324	1.379	1.41	2.67	no
PANSAA	1.357	1.381	1.362	1.306	1.352	1.36	1.361	1.322	1.377	1.406	5.44	no
PANSAA	1.37	1.378	1.359	1.302	1.36	1.357	1.358	1.321	1.377	1.395	5.44	no

QUTJAR	1.363	1.364	1.351	1.305	1.36	1.348	1.348	1.314	1.372	1.411	3.84	no
QUTJEV	1.408	1.343	1.339	1.321	1.379	1.349	1.342	1.295	1.389	1.394	7.74	no
QUTKEW	1.376	1.431	1.319	1.338	1.364	1.349	1.349	1.337	1.375	1.373	6.58	yes
RIGMEA	1.363	1.374	1.359	1.297	1.357	1.355	1.349	1.31	1.378	1.402	4.64	no
RIGMIE	1.373	1.39	1.362	1.306	1.372	1.362	1.361	1.326	1.389	1.418	4.38	no
ROPMIU	1.37	1.397	1.376	1.308	1.365	1.358	1.37	1.327	1.378	1.409	3.11	no
ROPMIU	1.373	1.391	1.369	1.301	1.376	1.352	1.364	1.32	1.383	1.405	3.11	no
SEQTUF	1.367	1.389	1.363	1.309	1.364	1.358	1.374	1.315	1.375	1.407	3.45	no
SEQTUF	1.372	1.387	1.358	1.301	1.36	1.362	1.365	1.313	1.39	1.407	3.45	no
SEQVAN	1.386	1.381	1.347	1.316	1.366	1.356	1.371	1.311	1.38	1.395	5.52	no
SIXZUW	1.37	1.391	1.348	1.31	1.357	1.353	1.357	1.314	1.374	1.403	5.98	yes
SIXZUW	1.388	1.401	1.346	1.31	1.356	1.344	1.355	1.318	1.365	1.393	5.98	yes
SIYBAF	1.356	1.379	1.337	1.316	1.342	1.363	1.355	1.307	1.378	1.399	5.54	yes
SIYBAF	1.366	1.383	1.347	1.303	1.354	1.347	1.353	1.311	1.37	1.403	5.54	yes
SOLCOM	1.375	1.389	1.362	1.31	1.36	1.354	1.371	1.314	1.378	1.404	3.32	no
SOLCOM01	1.364	1.382	1.363	1.303	1.362	1.347	1.361	1.307	1.381	1.4	3.83	no
SOLDAZ	1.369	1.387	1.369	1.297	1.358	1.36	1.36	1.326	1.38	1.407	2.73	no
SOLDAZ01	1.363	1.379	1.368	1.306	1.358	1.35	1.355	1.324	1.379	1.392	3.13	no
TINYIZ	1.371	1.386	1.365	1.309	1.359	1.365	1.372	1.321	1.383	1.41	3.49	no
TUDLOT	1.357	1.384	1.344	1.313	1.347	1.363	1.349	1.315	1.379	1.4	4.5	no
UDIJEX	1.362	1.378	1.316	1.324	1.35	1.356	1.354	1.312	1.376	1.408	4.95	no
UDIJEX	1.375	1.379	1.338	1.303	1.356	1.357	1.344	1.31	1.389	1.387	4.95	no
UWAMEM	1.373	1.394	1.356	1.307	1.363	1.355	1.358	1.316	1.382	1.404	5.89	no
UWAMEM01	1.359	1.384	1.347	1.319	1.364	1.364	1.359	1.31	1.395	1.394	6.62	no
UWAMEM01	1.374	1.395	1.355	1.297	1.358	1.354	1.361	1.326	1.389	1.405	6.62	no
UWAMUC	1.368	1.38	1.36	1.31	1.359	1.361	1.351	1.313	1.377	1.415	4.23	no
VEWFIP	1.356	1.384	1.35	1.307	1.356	1.359	1.356	1.329	1.373	1.405	4.48	no
VEWFIP	1.357	1.38	1.351	1.314	1.36	1.362	1.358	1.317	1.381	1.406	4.48	no
VEWFIP	1.358	1.386	1.355	1.296	1.348	1.36	1.361	1.312	1.379	1.407	4.48	no

VEWFIP	1.362	1.384	1.358	1.3	1.36	1.353	1.358	1.317	1.375	1.405	4.48	no
VIGVEM	1.357	1.389	1.345	1.309	1.344	1.355	1.35	1.312	1.386	1.394	6.2	yes
VITMES	1.357	1.383	1.361	1.296	1.357	1.358	1.359	1.314	1.377	1.404	4.03	no
VOFVOE	1.369	1.387	1.358	1.319	1.354	1.349	1.36	1.331	1.372	1.401	7.46	no
WUQNAZ	1.372	1.383	1.361	1.305	1.36	1.358	1.36	1.325	1.383	1.412	3.77	no
XIVBOV	1.35	1.377	1.325	1.331	1.342	1.363	1.344	1.305	1.377	1.417	8.77	no
**$AdeH_2^{2+}$**												
ZUKTEH	1.387	1.381	1.343	1.298	1.348	1.338	1.37	1.33	1.378	1.403	1.95	no
ZUKTEH	1.388	1.369	1.341	1.285	1.39	1.347	1.343	1.327	1.355	1.401	1.95	no
ADENDH	1.358	1.377	1.361	1.302	1.348	1.372	1.335	1.321	1.376	1.4	3.6	no
ADENDH02	1.356	1.379	1.358	1.308	1.349	1.375	1.338	1.327	1.374	1.409	2.41	no
ADENDH03	1.354	1.378	1.355	1.299	1.345	1.37	1.329	1.322	1.376	1.395	2.9	no
ADENDH04	1.359	1.378	1.364	1.308	1.353	1.377	1.34	1.326	1.376	1.401	2.65	no
ADENSL	1.365	1.371	1.361	1.295	1.359	1.371	1.334	1.327	1.381	1.411	5.5	no
AHEWIT	1.364	1.369	1.344	1.305	1.36	1.359	1.338	1.327	1.365	1.409	3.19	no
AHEWOZ	1.381	1.393	1.321	1.313	1.364	1.348	1.353	1.313	1.371	1.395	3.96	no
AHEWUF	1.367	1.365	1.349	1.295	1.352	1.358	1.331	1.317	1.377	1.402	3.19	no
BIDRUB10	1.35	1.373	1.364	1.301	1.347	1.379	1.318	1.323	1.382	1.418	2.6	no
BIDRUB11	1.364	1.37	1.363	1.306	1.357	1.376	1.325	1.324	1.383	1.416	2.41	no
BOTSEJ	1.358	1.379	1.361	1.306	1.353	1.369	1.329	1.321	1.377	1.414	3.06	no
DECVOZ	1.361	1.373	1.367	1.3	1.346	1.367	1.339	1.315	1.375	1.401	2.38	no
DECVUF	1.365	1.361	1.364	1.296	1.36	1.369	1.334	1.321	1.382	1.398	2.08	no
DUJHUN	1.361	1.374	1.36	1.293	1.345	1.364	1.324	1.319	1.375	1.406	4.54	no
DUMYIU	1.356	1.374	1.358	1.298	1.352	1.364	1.32	1.321	1.376	1.41	3.95	no
EFADAS	1.365	1.368	1.363	1.308	1.358	1.379	1.328	1.331	1.386	1.411	4.28	no
EFADAS	1.376	1.379	1.362	1.308	1.358	1.37	1.338	1.324	1.379	1.41	4.28	no
FOMXUB	1.353	1.369	1.364	1.297	1.348	1.373	1.325	1.324	1.37	1.408	2.66	no
GADWEO	1.36	1.381	1.368	1.304	1.354	1.373	1.347	1.314	1.379	1.414	3.55	yes
PAVHOM	1.371	1.375	1.347	1.299	1.352	1.37	1.309	1.316	1.384	1.401	4.12	no

Table S7. The bond length [Å] and angles [deg] in the model **1p.**

			**Bond length [Å]**				
W1	C1	Double	2.2076	N1	Cu1	Single	2.1272
W1	C2	Single	2.1861	N3	Cu1	Single	2.0135
W1	C3	Single	2.2119	N4	Cu1	Single	1.9744
W1	C4	Single	2.1532	N5	Cu1	Single	1.9739
W1	C5	Single	2.1580	C6	N6	Triple	1.1663
W1	C6	Single	2.2537	C7	N7	Triple	1.1659
W1	C7	Single	2.2107	C8	N8	Triple	1.1409
W1	C8	Single	2.1983	N11	C12	Single	1.3118
Cu1	N2	Single	1.9884	C12	N13	Aromatic	1.3673
Cu1	N1	Single	2.1272	C12	C20	Aromatic	1.4086
Cu1	N3	Single	2.0135	N13	C14	Aromatic	1.3718
Cu1	N4	Single	1.9744	C14	N15	Aromatic	1.3102
Cu1	N5	Single	1.9739	N15	C16	Aromatic	1.3643
C1	N1	Double	1.2333	C16	N17	Single	1.3583
C2	N2	Triple	1.1448	C16	C20	Aromatic	1.3784
C3	N3	Triple	1.1582	N17	C18	Single	1.3560
C4	N4	Triple	1.1311	C18	N19	Double	1.3170
C5	N5	Triple	1.1354	N19	C20	Single	1.3834
			**Angle [°]**				
C1	W1	C2	76.15	N1	Cu1	N4	100.89
C1	W1	C3	75.57	N1	Cu1	N5	121.14
C1	W1	C4	75.93	N3	Cu1	N4	84.93
C1	W1	C5	83.30	N3	Cu1	N5	130.62
C1	W1	C6	137.89	N4	Cu1	N5	94.56
C1	W1	C7	138.39	W1	C1	N1	173.27
C1	W1	C8	133.73	W1	C2	N2	175.16
C2	W1	C3	85.24	W1	C3	N3	171.44
C2	W1	C4	152.07	W1	C4	N4	174.44
C2	W1	C5	97.83	W1	C5	N5	175.87
C2	W1	C6	72.25	C1	N1	Cu1	154.53
C2	W1	C7	144.10	Cu1	N2	C2	163.47
C2	W1	C8	89.12	C3	N3	Cu1	151.15
C3	W1	C4	87.08	C4	N4	Cu1	167.45
C3	W1	C5	157.30	C5	N5	Cu1	163.35
C3	W1	C6	74.75	W1	C6	N6	177.71
C3	W1	C7	94.03	W1	C7	N7	177.10
C3	W1	C8	147.53	W1	C8	N8	177.88
C4	W1	C5	79.73	N11	C12	N13	121.22
C4	W1	C6	130.99	N11	C12	C20	125.61
C4	W1	C7	63.22	N13	C12	C20	113.17
C4	W1	C8	111.02	C12	N13	C14	124.31
C5	W1	C6	127.67	N13	C14	N15	124.59

C5	W1	C7	96.35	C14	N15	C16	111.84
C5	W1	C8	55.17	N15	C16	N17	126.53
C6	W1	C7	72.96	N15	C16	C20	127.80
C6	W1	C8	73.08	N17	C16	C20	105.67
C7	W1	C8	72.49	C16	N17	C18	106.62
N2	Cu1	N1	106.57	N17	C18	N19	113.58
N2	Cu1	N3	85.44	C18	N19	C20	103.34
N2	Cu1	N4	152.50	C12	C20	C16	118.29
N2	Cu1	N5	72.73	C12	C20	N19	130.92
N1	Cu1	N3	107.27	C16	C20	N19	110.80

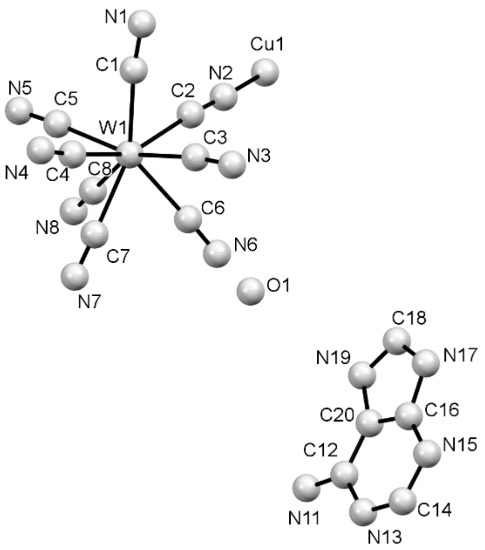

Figure S10. Asymmetric unit in the **1p** model.

Although the **1p** model assumes different crystallographic cell (*P*-1) compared to the model **1** from the SC XRD data (*C*2), it reproduce decently the overall coordination skeleton of the $\{Cu^{II}[M^{V}(CN)_8]\}^-$ bilayer, the interbilayer separation and the role of $AdeH^+$ blocks in the construction of the molecular architecture of **1**. For details see the comparison in Table S8. We consider the model **1** to be more reliable compared to **1p**, where the dimeric adenine synthon was prepared and introduced with constrained geometry and rotational degrees of freedom, according to the structural data from the CSD. However, we appreciate the latter one in the structural description, having in mind the natural limitation of this approach. In the description of supramolecular contacts we focus on the model **1**.

Table S8 The most important intermetallic, intralayer and interlayer distances in structural models **1** and **1p** and their comparison with the previously reported compounds (single crystal measurements).

	**1**	**1p**
	(a) Average intra-layer distances (Å)	
Cu_{eq}-W[a]	5.24	5.24
Cu_{ax}-W	5.44	5.46
Cu-Cu[b]	6.89	6.90
W-W[b]	7.81	7.81
	(b) Average inter-layer distances (Å)	
Cu···Cu[c]	**16.89**	**16.84**
Cu···W[c]	**15.64**	**15.60**
W···W[c]	**14.44**	**14.41**
W_{planes}[d]	**13.92**	**13.90**
Cu_{planes}[d]	**16.46**	**16.40**

[a] Average distance between central and 4 adjacent atoms
[b] Average distance between central and 8 adjacent atoms
[c] Average distance between central atom from 1st layer and 2 nearest atoms from 2nd layer
[d] Distance between 2 planes determined by position of corresponding atoms from 1st and 2nd layers

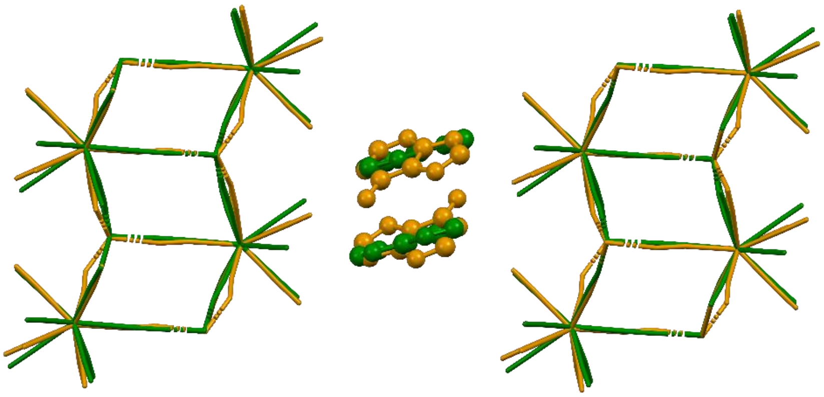

(a)

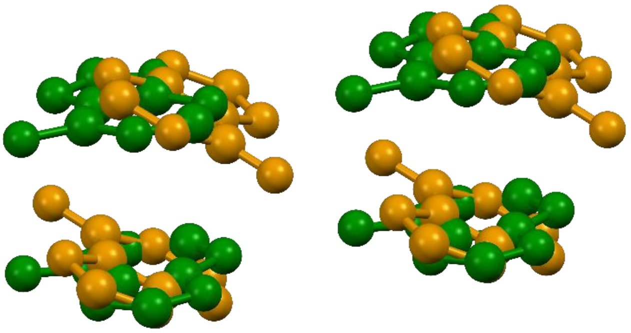

(b)

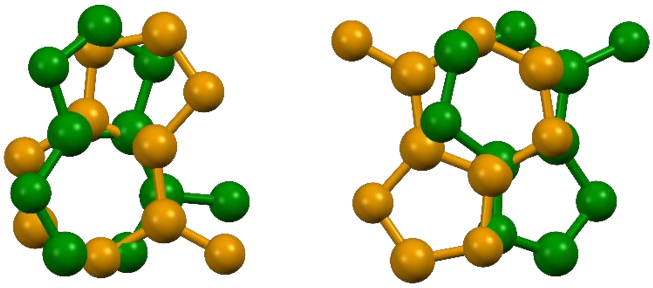

(c)

Figure S11. Overlap of the molecular architectures according to the structural models **1** (green) and **1p** (yellow). The coordination backbones are topologically identical and differ slightly only in some angles and bond lengths. The gravity centers of $AdeH^+$ cations in both models are located close to each other, however, the individual orientation of molecules and mutual orientation between molecules differ significantly.

Magnetic studies

The magnetic measurements were carried out with the MPMS XL magnetometer from Quantum Design. The single crystals were oriented along *b* crystallographic axis and attached to plastic plate with Apiezon M grease (Figure S12). The obtained single crystals were small (0.36 mm x 0.7 mm of in-plane dimensions with thickness below 0.1 mm), which caused difficulties with ideal alignment. In consequence, a small misalignment was present, which is responsible for small blurriness of the magnetic signal. However, all the crystals are aligned along b crystallographic axis and the magnetization is a pseudo-vector which does not change under the inversion operation, therefore all magnetic analysis is still valid. The magnetic measurements were performed in three orthogonal orientations: $a^*||H$, $b||H$ and $c||H$ (where a^*,b, c are the crystallographic directions and H is the applied filed). The dc magnetic susceptibilities were measured as a function of temperature in temperature range 2.0 K – 150.0 K under an applied field of 500 Oe. The temperature dependences of zero-field-cooled (ZFC) and field-cooled (FC) were measured in temperature range 2.0 K – 50.0 K with applied field of 50 Oe. Isothermal magnetization measurements were collected at 2.0 K in magnetic field range -7.0 T to 7.0 T. The angle-resolved susceptibility measurements were carried out around three orthogonal axes: a^*, b and c with Quantum Design Horizontal Sample Rotator at 2.0 K under an applied field of 1 kOe. All magnetic measurements were performed for the same batch of single crystals. The background signal from the rotator was subtracted with the Automatic Background Subtraction (ABS) option. The diamagnetic corrections were estimated by fitting experimental data.

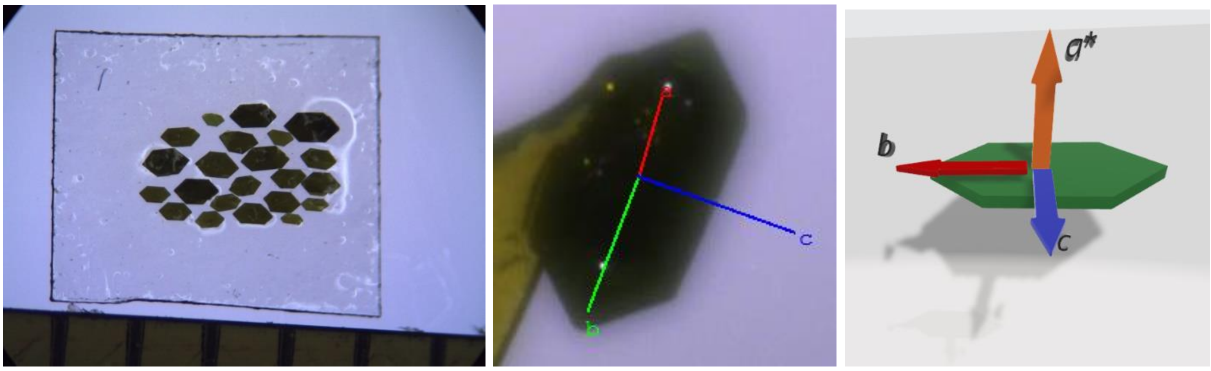

Figure S12. Sample preparation for the monocrystalline magnetic measurements and the results of single crystal indexing for **1**.

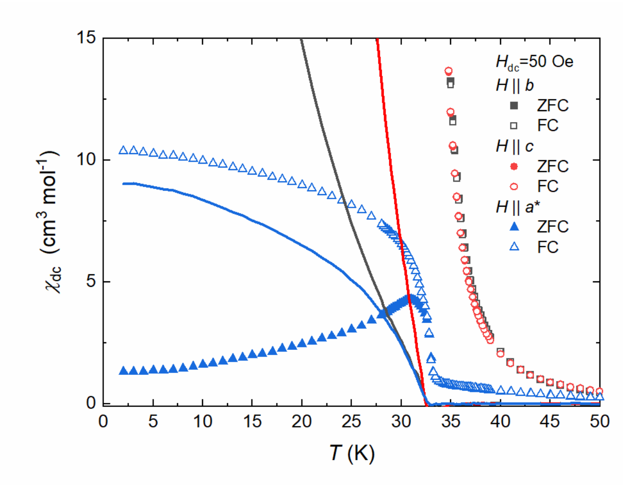

Figure S13. The ZFC/FC measurements for **1** along the crystallographic directions *a**, *b* and *c* (the small values range of χ_{dc} was focused on). The solid lines show the difference between the FC and ZFC data.

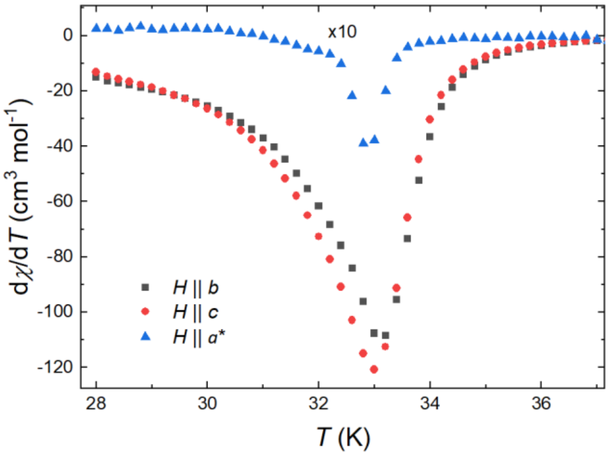

Figure S14. The first derivative of ZFC susceptibility for **1** along the crystallographic directions *a**, *b* and *c*.

Estimation of the diamagnetic contributions

The raw data of the temperature dependence of magnetization were recalculated using the sample mass of 0.000131 g and the molar mass per unit cell of 2366.6 g mol^{-1} to yield the molar magnetic susceptibilities in three orthogonal crystallographic directions a^*, b, c. The first task was that of determining the diamagnetic contribution to the susceptibility signal. It was performed for each direction separately. The molar susceptibility data in the temperature range of 50 – 150 K were modeled by the following function

$$\chi_\alpha = \frac{c_1}{T} + \frac{c_{2\alpha}}{T^2} + \frac{c_{3\alpha}}{T^3} + \chi_{0\alpha} \tag{S1}$$

where $\alpha = a^*, b, c$. The first three terms represent the high-temperature expansion, while the last term is the sought-for diamagnetic correction. The value of constant c_1 was fixed at 3.0 emu K mol^{-1} corresponding to the Curie-Weiss constant for four Cu(II) ions with S_{Cu}=1/2 and g_{Cu}=2.0, and four W(V) ions with S_W=1/2 and g_W=2.0. Figure S15 shows the results of this analysis.

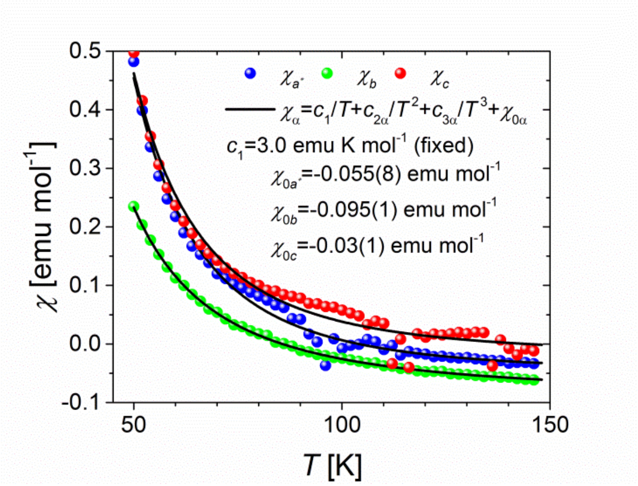

Figure S15. Molar magnetic susceptibilities measured for three orthogonal crystallographic directions (symbols). The best fit curves of Eq. (S1) to the experimental data yielding the diamagnetic contributions. Note that above plot shows data calculated per unit cell (four times larger than per formula).

The diamagnetic contributions were thus estimated to amount to -0.055(8), -0.095(1), and -0.03(1) emu mol^{-1} for the a^*, b, and c crystallographic directions, respectively. With the measured data we are not able to determine the full tensor of the diamagnetic contribution. Therefore, in view of the angle resolved measurements we decided to estimate the average value of the diamagnetic correction. To this end an analogous fit of the function in Eq. (S1) was fitted in the temperature range of 50 – 150 K to the raw molar magnetic susceptibility of the powder sample calculated as $\chi_{avg} = (\chi_{a^*} + \chi_b + \chi_c)/3$. Figure S16 shows the result of this procedure. The averaged diamagnetic correction was thus estimated to amount -0.059(5) emu mol^{-1} which correspond perfectly with the estimated value of the trace of the diamagnetic contribution tensor: $(\chi_{0a^*} + \chi_{0b} + \chi_{0c})/3 = -0.060(6)$ emu mol^{-1} per unit cell.

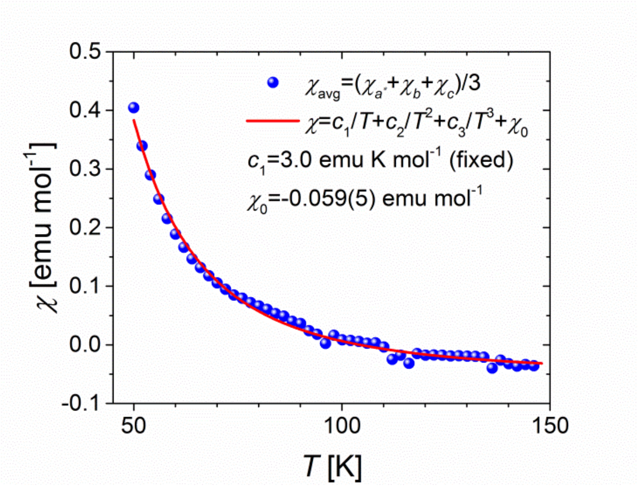

Figure S16. Temperature dependence of the powder raw magnetic susceptibility calculated using the values for the three orthogonal crystallographic directions (symbols). The best-fit curve of the function in Eq. (S1) yielding the averaged diamagnetic contribution (solid line). Note that above plot shows data calculated per unit cell.

Trigonometric polynomial expansion for the angle-resolved data

The quality of fit of the angle-resolved susceptibility data may be improved by adding to the present model other components of the four-rank tensor $\kappa_{\alpha\beta\mu\nu}$ as well as by extending the magnetization expansion in Eq. (1) to even higher orders. This is corroborated by the trigonometric polynomial approximation defined by

$$S_n(\theta) = \frac{a_0}{2} + a_n \cos(n\theta) + \sum_k^{n-1} [a_k \cos(k\theta) + b_k \sin(k\theta)]\,, \tag{S2}$$

where

$$\begin{aligned} a_k &= \frac{1}{m}\sum_{j=0}^{2m-1} \chi_j \cos(k\theta_j) \quad \text{for each } k = 0,1,\ldots,n, \\ b_k &= \frac{1}{m}\sum_{j=0}^{2m-1} \chi_j \sin(k\theta_j) \quad \text{for each } k = 0,1,\ldots,n-1, \end{aligned} \tag{S3}$$

where n is the approximation order, and $2m$=72 is the number of equally spaced experimental points. Indeed, Figure S17 shows the results of this approximation with n=10 applied to the susceptibility data, while Figure S18 provides the plots of the corresponding expansion coefficients (a_k, b_k). The agreement is close to perfect. Figure S17 indicates that the dominating

contribution comes from the terms comprising even multiples of the angle variable (even k). This is consistent with the fact that all odd-rank tensors in the expansion in Eq. (1) should vanish, unless there is some residual field present during the susceptibility measurement. This may be the case for the data detected by rotation around the hard magnetization direction ($\boldsymbol{a}^*$ crystallographic axis), as coefficients a_1 and b_1 reveal here slightly enhanced values. Although the improvement of the fit is in principle possible, it is by no means clear which components of the higher-rank tensors should be retained and which of them may be neglected. Taking all of them into account will quickly lead to the overparametrization of the optimization problem, as the number of components grows exponentially fast (the number of components of the n-rank tensor amounts to 3^n). Therefore, this issue constitutes a research problem in itself and we intend to address it in some future work.

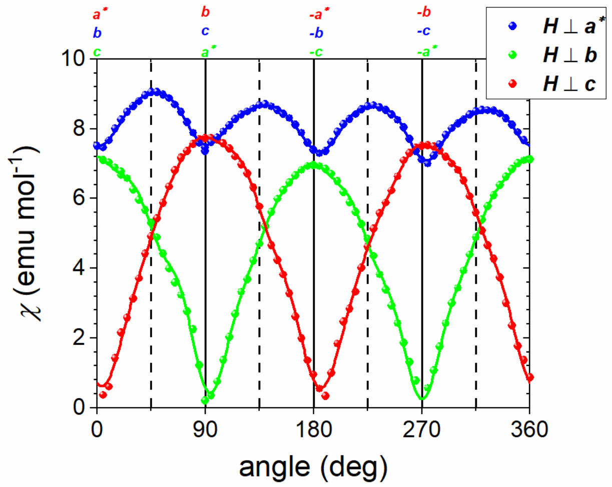

Figure S17. Trigonometric polynomial approximation applied to the angle-resolved susceptibility data.

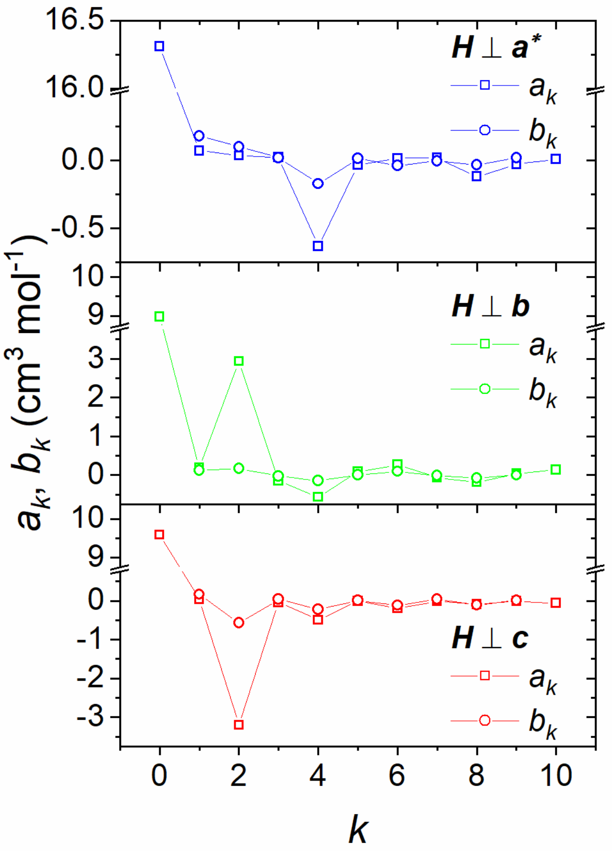

Figure S18. Expansion coefficients corresponding to the trigonometric polynomial approximation of the angle-resolved susceptibility data given in Figure S17.

Scaling analysis

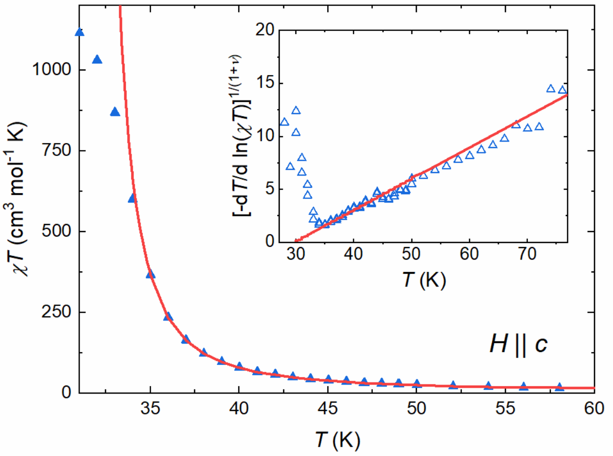

Figure S19. Critical scaling analysis of χT vs. T for $H \| c$. Insert shows the indication of Berezinskii-Kosterlitz-Thouless transition for $H \| c$. The red solid line shows the best fit to $\chi T = a_{\chi} e^{b_{\chi}(T-T_{BKT})^{-\nu}}$.

References

(1) Matoga, D.; Szklarzewicz J.; Mikuriya, M. $[PPh_4]_3[W(CN)_7(O_2)]\cdot 4H_2O$ as the Representative of the $[M(L)_7(LL)]$ Class for Nine-Coordinate Complexes. *Inorg. Chem.* **2006,** *45*, 7100–7104.

(2) Podgajny, R.; Korzeniak, T.; Bałanda, M.; Wasiutyński, T.; Errington, W.; Kemp, T. J.; Alcock, N. W.; Sieklucka, B. 2-D Soft Ferromagnet Based on $[W^{V}(CN)_8]^{3-}$ and Cu^{II} with a T_c of 34 K. *Chem. Commun.* **2002,** 1138–1139.

(3) Korzeniak, T.; Podgajny, R.; Alcock, N. W.; Lewiński, K.; Bałanda, M.; Wasiutyński, T.; Sieklucka, B. A New Family of Magnetic 2-D Coordination Polymers Based on $[M^{V}(CN)_8]^{3-}$ (M = Mo, W) and pre-programmed Cu^{2+} centres. *Polyhedron* **2003,** *22*, 2183–2190.

(4) Kaneko, S.; Tsunobuchi, Y.; Sakurai, S.; Ohkoshi, S. Two-Dimensional Metamagnet Composed of a Cesium Copper Octacyanotungstate. *Chem. Phys. Lett.*, **2007**, *446*, 292–296.

(5) Stefańczyk, O.; Majcher, A. M.; Rams, M.; Nitek, W.; Kozieł, M.; Łasocha, W.; Sieklucka, B. Incorporation of Guanidinium Ions in Cu^{II}-$[M^{V}(CN)_8]^{3-}$ Double-Layered Magnetic Systems. Dalton Trans. **2013,** *42*, 5042–5046.

(6) Konieczny, P.; Pełka, R.; Czernia, D., Podgajny, R. Rotating Magnetocaloric Effect in an Anisotropic Two-Dimensional $Cu^{II}[W^{V}(CN)_8]^{3-}$ Molecular Magnet with Topological Phase Transition: Experiment and Theory. *Inorg. Chem.* **2017,** *56*, 11971–11980.

(7) Kabsch, W. XDS. *Acta Cryst.* **2010,** *D66*, 125–132.

(8) Krug, M.; Weiss, M. S.; Heinemann, U.; Mueller, U. XDSAPP: a Graphical User Interface for the Convenient Processing of Diffraction Data Using XDS. *J. Appl. Crystallogr.* **2012,** *45*, 568–572.

(9) CrysAlis RED and CrysAlis CCD. Oxford Diffraction Ltd., Abingdon, Oxfordshire, England, 2000.

(10) Sheldrick, G. M. Crystal structure refinement with SHELXL. *Acta Cryst.* **2015,** *C71*, 3–8.

(11) Farrugia, L. J. *WinGX suite for small-molecule single-crystal crystallography. J Appl. Crystallogr.* **1999,** *32*, 837–838.

(12) Altomare, A.; Cuocci, C.; Giacovazzo, C.; Moliterni, A.; Rizzi, R.; Corriero N.; Falcicchio, A. EXPO2013: a Kit of Tools for Phasing Crystal Structures from Powder Data. *J. Appl. Cryst.* **2013,** *46*, 1231–1235.

(13) Favre-Nicolin, V.; Cerny, R. FOX, `Free Objects for Crystallography': a Modular Approach to Ab Initio Structure Determination from Powder Diffraction. *J. Appl. Cryst.* **2002,** *35*, 734–743.

(14) Groom, C. R.; Bruno, I. J.; Lightfoot, M. P.; Ward, S. C. The Cambridge Structural Database. *Acta Cryst.* **2016,** *B72*, 171–179

(15) Petricek, V., Dusek, M.; Palatinus, L. Crystallographic Computing System JANA2006: General features Z. *Kristallogr.* **2014,** *229*, 345-352.

(16) Sridhar, B.; Ravikumar, K.; Varghese, B. Supramolecular Hydrogen-Bonded Networks in Adeninediium Hemioxalate Chloride and Adeninium Semioxalate Hemi(oxalic acid) Monohydrate, *Acta Cryst.* **2009,** *C65*, o202–o206.

(17) Hingerty, B. E.; Einstein, J. R.; Wei, C. H. Structure of bisadeninium dinitrate monohydrate, $(C_5H_6N_5^{+}\cdot NO^{3-})_2\cdot H_2O$. *Acta Cryst.* **1981,** *B37*, 140–147.

(18) Langer, V.; Huml, K. The crystal and molecular structure of adeninium sulphate, $C_5H_5N_5\cdot H_2SO_4$. *Acta Cryst.* **1978,** *B34*, 1157–1163.

(19) Christensen, J. J.; Rytting, J. H.; Izatt, R. M. Thermodynamic pK, AH0, AS0, and ACP° Values for Proton Dissociation from Several Purines and Their Nucleosides in Aqueous Solution. *Biochemistry*, **1970,** *9*, 4907–4913.

(20) Tureček, F.; Chen, X. Protonated Adenine: Tautomers, Solvated Clusters, and Dissociation Mechanisms. *J. Am. Soc. Mass Spectrom.* **2005,** *16*, 1713–1726.

(21) Pasquali, S.; Frezza, E.; Barroso da Silva, F. L. Coars-Grained Dynamic RNA Titration Simulations. *Interface Focus* **2019**, *9*, 20180066.
